# Nonlinear impulsive differential and integral inequalities with nonlocal jump conditions

**DOI:** 10.1186/s13660-018-1762-3

**Published:** 2018-07-13

**Authors:** Zhaowen Zheng, Yingjie Zhang, Jing Shao

**Affiliations:** 1 School of Mathematical Sciences, Qufu Normal University, Qufu, P.R. China; 2 Department of Mathematics, Jining University, Qufu, P.R. China; 3 Institute of Applied Physics and Computational Mathematics, Beijing, P.R. China

**Keywords:** Nonlinear impulsive, Differential and integral inequalities, Nonlocal intergal jump conditions, Riemann–Liouville fractional derivative

## Abstract

Some new nonlinear impulsive differential and integral inequalities with nonlocal integral jump conditions are presented in this paper. Using the method of mathematical induction, we obtain a new upper bound estimation of certain differential and integral inequalities; these inequalities have both nonlocal integral jump and weakly singular kernels. Finally, we give two examples of these inequalities in estimating solutions of certain equations with Riemann–Liouville fractional integral conditions.

## Introduction

As is well known, impulsive differential and impulsive integral inequalities play a fundamental part in the study of theory of impulsive equations (see [1–4]). Recently, a lot of experts studied the global existence, uniqueness, bounded-ness, stability, oscillation and other properties of different impulsive inequalities (see [5–18]). For example, in [1], Lakshmikanthan investigated an impulsive differential inequality given as Theorem 1.1.

Let $0\leq t_{0}< t_{1}< t_{2}<\cdots$ be a sequence, $\lim_{k\rightarrow \infty}t_{k}=\infty$, $\mathbb{R}_{+}=[0, +\infty)$. For $I\subset\mathbb {R}$, we define the following set of functions:

$PC(\mathbb{R}_{+}, I)$ = {$u:\mathbb{R}_{+}\rightarrow I$; $u(t)$ is continuous for $t\neq t_{k}$, $u(0^{+})$, $u(t_{k}^{+})$, $u(t_{k}^{-})$ exist, and $u(t)$ is left-continuous at $t_{k}$, $k=1, 2, \ldots$};

$PC^{1}(\mathbb{R}_{+}, I)$ = {$u\in PC(\mathbb{R}_{+}, I)$; $u'(t)$ is continuous for $t\neq t_{k}$, $u'(0^{+})$, $u'(t_{k}^{+})$, $u'(t_{k}^{-})$ exist, and $u'(t)$ is left-continuous at $t_{k}$, $k=1, 2, \ldots$}.

### Theorem 1.1

*Assume that*:

(H_0_): *the sequence* $\{t_{k}\}$ *satisfies* $0\leq t_{0}< t_{1}< t_{2}<\cdots$, $\lim_{k\rightarrow\infty}t_{k}=\infty$;
(H_1_): $m\in PC^{1}[\mathbb{R}_{+}, \mathbb{R}]$ *and* $m(t)$ *is left*-*continuous at* $t_{k}$, $k=1, 2, \ldots $ ;
(H_2_): *for* $k=1, 2, \ldots$ , $t\geq t_{0}$,
  1.1 $$\begin{aligned}& m'(t)\leq p(t)m(t)+q(t), \quad t\neq t_{k}, \end{aligned}$$
  1.2 $$\begin{aligned}& m\bigl(t_{k}^{+}\bigr)\leq d_{k}m(t_{k})+b_{k}, \end{aligned}$$
  *where* $p, q\in C[\mathbb{R}_{+}, \mathbb{R}]$, $d_{k}\geq0$ *and* $b_{k}$ ($k=1, 2, \ldots$) *are constants*. *Then*
  1.3 $$\begin{aligned} m(t) \leq& m(t_{0})\prod_{t_{0}< t_{k}< t}d_{k}e^{\int^{t}_{t_{0}}p(\xi )\,d\xi }+ \sum_{t_{0}< t_{k}< t}\biggl(\prod_{t_{k}< t_{j}< t}d_{j}e^{\int ^{t}_{t_{k}}p(\xi)\,d\xi} \biggr)b_{k} \\ &{}+ \int^{t}_{t_{0}}\prod_{s< t_{k}< t}d_{k}e^{\int^{t}_{s}p(\xi)\,d\xi }q(s) \,ds,\quad t\geq t_{0}. \end{aligned}$$

In [12], Thiramanus and Tariboon developed the impulsive inequalities with the following integral jump conditions:

1.4 $$ m\bigl(t_{k}^{+}\bigr)\leq d_{k}m(t_{k})+c_{k} \int_{t_{k}-\tau_{k}}^{t_{k}-\sigma _{k}}m(s)\,ds+b_{k},\quad k=1, 2, \ldots, $$

where $0\leq\sigma_{k}\leq\tau_{k}\leq t_{k}-t_{k-1}$. In [11], Liengtragulngam et al. generalized further results by replacing the integral jump conditions (1.2) by the following nonlocal jump conditions:

1.5 $$ m\bigl(t_{k}^{+}\bigr)\leq\frac{c_{k}}{\Gamma(\beta_{k})} \int ^{t_{k}}_{t_{k-1}}(t_{k}-s)^{\beta_{k}-1} m(s)\,ds+d_{k}m(t_{k})+b_{k}. $$

We note that a weak singular kernel is involved in the nonlocal jump conditions. They gave the estimation of $m(t)$ as follows.

### Theorem 1.2

*Let* (H_0_) *and* (H_1_) *be true*. *Suppose that* $p, q\in C [\mathbb{R}_{+}, \mathbb{R}]$ *and for* $k=1, 2, \ldots$ , $t\geq t_{0}$,

$$\textstyle\begin{cases} m'(t)\leq p(t)m(t)+q(t), \quad t\neq t_{k}, \\ m(t_{k}^{+})\leq\frac{c_{k}}{\Gamma(\beta_{k})}\int ^{t_{k}}_{t_{k-1}}(t_{k}-s)^{\beta_{k}-1} m(s)\,ds+d_{k}m(t_{k})+b_{k}, \end{cases} $$

*where* $c_{k}, d_{k}\geq0$, $\beta_{k}>0$ *and* $b_{k}$ ($k=1, 2, \ldots$) *are constants*. *Then*, *for all* $t\geq t_{0}$,

1.6 $$\begin{aligned} m(t) \leq& \biggl\{ m(t_{0})\prod_{t_{0}< t_{k}< t} \biggl(\frac{c_{k}}{\Gamma(\beta_{k})} \int^{t_{k}}_{t_{k-1}}(t_{k}-s)^{\beta _{k}-1}e^{\int^{s}_{t_{k-1}}p(\xi)\,d\xi} \,ds+d_{k}e^{\int ^{t_{k}}_{t_{k-1}}p(\xi)\,d\xi} \biggr) \\ &{}+\sum_{t_{0}< t_{k}< t} \biggl[\prod _{t_{0}< t_{j}< t} \biggl(\frac {c_{j}}{\Gamma(\beta_{j})} \int^{t_{j}}_{t_{j-1}}(t_{j}-s)^{\beta _{j}-1}e^{\int^{s}_{t_{j-1}}p(\xi)\,d\xi} \,ds+d_{j}e^{\int ^{t_{j}}_{t_{j-1}}p(\xi)\,d\xi} \biggr) \\ &{}\times\biggl(\frac{c_{k}}{\Gamma(\beta_{k})} \int^{t_{k}}_{t_{k-1}} \int ^{s}_{t_{k-1}}(t_{k}-s)^{\beta_{k}-1}q( \nu)e^{\int^{s}_{\nu}p(\xi)\,d\xi }\,d\nu \,ds \\ &{}+d_{k} \int^{t_{k}} _{t_{k-1}} q(s)e^{\int^{t_{k}}_{s}p(\xi)\,d\xi } \,ds+b_{k}\biggr) \biggr] \biggr\} e^{\int^{t}_{t_{l}}p(\xi)\,d\xi}+ \int^{t} _{t_{l}} q(s)e^{\int^{t}_{s}p(\xi)\,d\xi} \,ds, \end{aligned}$$

*where* $t_{l}=\max\{t_{k}:t\geq t_{k}, k=1, 2, \ldots\}$.

These results play fundamental roles in the global existence, uniqueness, stability and other properties of various linear impulsive differential and integral equations.

A lot of authors just study the qualitative properties of linear impulsive inequalities. However, most of the phenomena in the world do not change linearly, such as heart beat, blood pressure, and so on. Hence the nonlinear impulsive differential and integral theories are more accurate than linear impulsive theories in various aspects.

In this paper, we extend the theories of linear impulsive system to nonlinear impulsive inequalities with nonlocal jump conditions. We consider the following nonlinear inequality:

$$ m'(t)\leq p(t)m(t)+q(t)m^{\alpha}(t),\quad t\neq t_{k}, $$

with different nonlocal jump conditions, we give the upper bound estimation of the inequality, and an estimation of solutions of certain nonlinear equations is also involved.

For convenience, we give the following lemmas.

### Lemma 1.1

([9])

*Assume that* $a, b\in\mathbb{R}$, $p\geq 0$. *Then*

$$\bigl( \vert a \vert + \vert b \vert \bigr)^{p}\leq C_{p}\bigl( \vert a \vert ^{p}+ \vert b \vert ^{p}\bigr), $$

*where* $C_{p}=1$ *for* $0\leq p\leq1$, *and* $C_{p}=2^{p-1}$ *for* $p>1$.

### Lemma 1.2

*Let* $\{a_{n}\}$, $\{b_{n}\}$ *be two sequences of numbers*. *Then we have*

$$ \Biggl(\sum_{k=1}^{n-1}\prod _{j=k+1}^{n-1}a_{j}b_{k} \Biggr)a_{n}+b_{n}=\sum_{k=1}^{n} \Biggl[\prod_{j=k+1}^{n}a_{j} \Biggr]b_{k}. $$

## Nonlinear impulsive inequalities with nonlocal jump conditions

In this section, we present and prove some new nonlinear impulsive differential and integral inequalities with nonlocal jump conditions. Let $t_{l}=\max\{t_{k}:t\geq t_{k}, k=1, 2, \ldots\}$.

### Theorem 2.1

*Let* (H_0_) *and* (H_1_) *hold*. *Suppose that* $p, q\in C[\mathbb{R}_{+}, \mathbb{R}]$ *and for* $k=1, 2, \ldots$ , $t\geq t_{0}$,

2.1 $$\begin{aligned}& m'(t)\leq p(t)m(t)+q(t)m^{\alpha}(t),\quad t \neq t_{k}, \end{aligned}$$

2.2 $$\begin{aligned}& m^{1-\alpha}\bigl(t_{k}^{+}\bigr)\leq \frac{c_{k}}{\Gamma(\beta_{k})} \int ^{t_{k}}_{t_{k-1}}(t_{k}-s)^{\beta_{k}-1} m^{1-\alpha}(s)\,ds+d_{k}m^{1-\alpha}(t_{k})+b_{k}, \end{aligned}$$

*where* $0<\alpha<1$, $c_{k}, d_{k}\geq0$, $\beta_{k}>0$ *and* $b_{k}$ ($k=1, 2, \ldots$) *are constants*. *Then*, *for* $t\geq t_{0}$, *we have*

2.3 $$\begin{aligned} m(t) \leq& \biggl\{ \biggl\{ m^{1-\alpha}(t_{0})\prod _{t_{0}< t_{k}< t}\biggl(\frac {c_{k}}{\Gamma(\beta_{k})} \int^{t_{k}}_{t_{k-1}}(t_{k}-s)^{\beta _{k}-1}e^{\int^{s}_{t_{k-1}}(1-\alpha)p(\xi)\,d\xi} \,ds \\ &{}+d_{k}e^{\int^{t_{k}}_{t_{k-1}}(1-\alpha)p(\xi)\,d\xi}\biggr) \\ &{}+\sum_{t_{0}< t_{k}< t}\biggl[\prod _{t_{k}< t_{j}< t}\biggl(\frac{c_{j}}{\Gamma (\beta_{j})} \int^{t_{j}}_{t_{j-1}}(t_{j}-s)^{\beta_{j}-1}e^{\int ^{s}_{t_{j-1}}(1-\alpha)p(\xi)\,d\xi} \,ds \\ &{}+d_{j}e^{\int^{t_{j}}_{t_{j-1}}(1-\alpha)p(\xi)\,d\xi}\biggr) \biggr] \\ &{}\times\biggl(\frac{c_{k}}{\Gamma(\beta_{k})}(1-\alpha) \int ^{t_{k}}_{t_{k-1}} \int^{s}_{t_{k-1}}(t_{k}-s)^{\beta_{k}-1}q( \nu)e^{\int ^{s}_{\nu}(1-\alpha)p(\xi)\,d\xi}\,d\nu \,ds \\ &{}+(1-\alpha)d_{k} \int^{t_{k}} _{t_{k-1}} q(s)e^{\int ^{t_{k}}_{s}(1-\alpha)p(\xi)\,d\xi} \,ds+b_{k}\biggr) \biggr\} e^{\int ^{t}_{t_{l}}(1-\alpha)(1-\alpha)p(\xi)\,d\xi} \\ &{}+(1-\alpha) \int^{t} _{t_{l}} q(s)e^{\int^{t}_{s}(1-\alpha)p(\xi)\,d\xi }\,ds\biggr\} ^{\frac{1}{1-\alpha}}. \end{aligned}$$

### Proof

Let

$$v(t)=m^{1-\alpha}(t). $$

Then applying (2.1), we can get

2.4 $$\begin{aligned} v'(t) =& (1-\alpha)m^{-\alpha}(t)m'(t) \\ \leq& (1-\alpha)m^{-\alpha}(t)\bigl[p(t)m(t)+q(t)m^{\alpha}(t)\bigr] \\ =& (1-\alpha)p(t)v(t)+(1-\alpha)q(t). \end{aligned}$$

The inequality (2.2) can be written as

2.5 $$ v\bigl(t_{k}^{+}\bigr)\leq\frac{c_{k}}{\Gamma(\beta_{k})} \int ^{t_{k}}_{t_{k-1}}(t_{k}-s)^{\beta_{k}-1} v(s)\,ds+d_{k}v(t_{k})+b_{k}. $$

Next, we set

2.6 $$\begin{aligned}& E_{k}=\frac{c_{k}}{\Gamma(\beta_{k})} \int ^{t_{k}}_{t_{k-1}}(t_{k}-s)^{\beta_{k}-1}e^{\int^{s}_{t_{k-1}}(1-\alpha )p(\xi)\,d\xi} \,ds+d_{k}e^{\int^{t_{k}}_{t_{k-1}}(1-\alpha)p(\xi)\,d\xi}, \end{aligned}$$

2.7 $$\begin{aligned}& \begin{aligned}[b] G_{k}&=\frac{c_{k}}{\Gamma(\beta_{k})} \int^{t_{k}}_{t_{k-1}} \int ^{s}_{t_{k-1}}(t_{k}-s)^{\beta_{k}-1}(1- \alpha)q(\nu)e^{\int^{s}_{\nu }p(\xi)\,d\xi}\,d\nu \,ds \\ &\quad {}+(1-\alpha)d_{k} \int^{t_{k}} _{t_{k-1}} q(s)e^{\int ^{t_{k}}_{s}(1-\alpha)p(\xi)\,d\xi} \,ds+b_{k}, \end{aligned} \end{aligned}$$

then (2.3) reduces to

2.8 $$\begin{aligned} m(t) \leq& \biggl\{ \biggl\{ m^{1-\alpha}(t_{0})\prod _{t_{0}< t_{k}< t}E_{k}+ \sum_{t_{0}< t_{k}< t} \biggl[\prod_{t_{k}< t_{j}< t}E_{j} \biggr]G_{k} \biggr\} e^{\int^{t}_{t_{l}}(1-\alpha)p(\xi)\,d\xi} \\ &{}+(1-\alpha) \int ^{t} _{t_{l}} q(s)e^{\int^{t}_{s}(1-\alpha)p(\xi)\,d\xi}\,ds \biggr\} ^{\frac {1}{1-\alpha}}. \end{aligned}$$

By the definition of $v(t)$, we just need to prove

2.9 $$\begin{aligned} v(t) \leq& \biggl\{ v(t_{0})\prod_{t_{0}< t_{k}< t}E_{k}+ \sum_{t_{0}< t_{k}< t} \biggl[\prod _{t_{k}< t_{j}< t}E_{j} \biggr]G_{k} \biggr\} e^{\int ^{t}_{t_{l}}(1-\alpha)p(\xi)\,d\xi} \\ &{}+(1-\alpha) \int^{t} _{t_{l}} q(s)e^{\int^{t}_{s}(1-\alpha)p(\xi)\,d\xi} \,ds. \end{aligned}$$

We prove it by induction. For $t\in[t_{0}, t_{1}]$, inequality (2.4) can be written as

2.10 $$ \frac{d}{dt}\bigl[v(t)e^{-\int^{t}_{t_{0}}(1-\alpha)p(\xi)\,d\xi}\bigr]\leq (1-\alpha)q(t) e^{-\int^{t}_{t_{0}}(1-\alpha)p(\xi)\,d\xi}. $$

Integrating (2.10) from $t_{0}$ to *t*, for $t\in[t_{0}, t_{1}]$, we have

2.11 $$ v(t)\leq v(t_{0})e^{\int^{t}_{t_{0}}(1-\alpha)p(\xi)\,d\xi}+(1-\alpha ) \int^{t}_{t_{0}} q(s) e^{\int^{t}_{s}(1-\alpha)p(\xi)\,d\xi} \,ds. $$

Hence (2.9) is valid on $[t_{0}, t_{1}]$. Assume that (2.9) holds for $t\in[t_{0}, t_{n}]$, for some integer $n>1$. Then, for $t\in[t_{n}, t_{n+1}]$, it follows from (2.4) and (2.11) that

2.12 $$ v(t)\leq v\bigl(t_{n}^{+}\bigr)e^{\int^{t}_{t_{n}}(1-\alpha)p(\xi)\,d\xi }+(1-\alpha) \int^{t}_{t_{n}} q(s) e^{\int^{t}_{s}(1-\alpha)p(\xi)\,d\xi} \,ds. $$

Applying (2.5) with (2.12), we get

2.13 $$\begin{aligned} v(t) \leq& \biggl(\frac{c_{n}}{\Gamma(\beta_{n})} \int ^{t_{n}}_{t_{n-1}}(t_{n}-s)^{\beta_{n}-1} v(s)\,ds+d_{n}v(t_{n})+b_{n} \biggr)e^{\int^{t}_{t_{n}}(1-\alpha)p(\xi)\,d\xi } \\ &{}+(1-\alpha) \int^{t}_{t_{n}} q(s) e^{\int^{t}_{s}(1-\alpha)p(\xi)\,d\xi} \,ds. \end{aligned}$$

By induction and (2.13), we get

2.14 $$\begin{aligned} v(t) \leq& \biggl\{ \frac{c_{n}}{\Gamma(\beta_{n})} \int ^{t_{n}}_{t_{n-1}}(t_{n}-s)^{\beta_{n}-1} \times \biggl\{ \biggl\{ v(t_{0})\prod_{t_{0}< t_{k}< s}E_{k}+ \sum_{t_{0}< t_{k}< s}\biggl[\prod_{t_{k}< t_{j}< s}E_{j} \biggr]G_{k} \biggr\} \\ &{}\times e^{\int^{s}_{t_{n-1}}(1-\alpha)p(\xi)\,d\xi}+(1-\alpha) \int ^{s}_{t_{n-1}} q(\nu) e^{\int^{s}_{\nu}(1-\alpha)p(\xi)\,d\xi}\,d\nu \biggr\} \,ds \\ &{}+d_{n} \biggl\{ \biggl\{ v(t_{0})\prod _{t_{0}< t_{k}< t_{n}}E_{k}+\sum_{t_{0}< t_{k}< t_{n} } \biggl[\prod_{t_{k}< t_{j}< t_{n}}E_{j} \biggr]G_{k} \biggr\} e^{\int^{t_{n}}_{t_{n-1}}(1-\alpha)p(\xi)\,d\xi} \\ &{}+(1-\alpha) \int^{t_{n}}_{t_{n-1}} q(s) e^{\int^{t_{n}}_{s}(1-\alpha )p(\xi)\,d\xi}\,ds \biggr\} +b_{n} \biggr\} \\ &{}\times e^{\int^{t}_{t_{n}}(1-\alpha)p(\xi)\,d\xi}+(1-\alpha) \int ^{t}_{t_{n}} q(s) e^{\int^{t}_{s}(1-\alpha)p(\xi)\,d\xi}\,ds \\ =& \biggl\{ \biggl(v(t_{0})\prod_{t_{0}< t_{k}< t_{n}}E_{k}+ \sum_{t_{0}< t_{k}< t_{n}}\biggl[\prod_{t_{k}< t_{j}< t_{n}}E_{j} \biggr]G_{k} \biggr) \frac{c_{n}}{\Gamma(\beta_{n})} \int^{t_{n}}_{t_{n-1}}(t_{n}-s)^{\beta _{n}-1} \\ &{}\times e^{\int^{s}_{t_{n-1}}(1-\alpha)p(\xi)\,d\xi} \,ds \\ &{}+\frac{c_{n}}{\Gamma(\beta_{n})} \int^{t_{n}}_{t_{n-1}} \int ^{s}_{t_{n-1}}(t_{n}-s)^{\beta_{n}-1}(1- \alpha)q(\nu)e^{\int^{s}_{\nu }(1-\alpha)p(\xi)\,d\xi}\,d\nu \,ds \\ &{}+ \biggl(v(t_{0})\prod_{t_{0}< t_{k}< t_{n}}E_{k}+ \sum_{t_{0}< t_{k}< t_{n}}\biggl[\prod_{t_{k}< t_{j}< t_{n}}E_{j} \biggr]G_{k} \biggr)d_{n}e^{\int ^{t_{n}}_{t_{n-1}}(1-\alpha)p(\xi)\,d\xi} \\ &{}+d_{n}(1-\alpha) \int^{t_{n}}_{t_{n-1}} q(s) e^{\int ^{t_{n}}_{s}(1-\alpha)p(\xi)\,d\xi} \,ds+b_{n} \biggr\} e^{\int ^{t}_{t_{n}}(1-\alpha)p(\xi)\,d\xi} \\ &{}+(1-\alpha) \int^{t}_{t_{n}} q(s) e^{\int^{t}_{s}(1-\alpha)p(\xi)\,d\xi}\,ds \\ =& \biggl\{ \biggl(v(t_{0})\prod_{t_{0}< t_{k}< t_{n}}E_{k}+ \sum_{t_{0}< t_{k}< t_{n}}\biggl[\prod_{t_{k}< t_{j}< t_{n}}E_{j} \biggr]G_{k} \biggr)E_{n} +G_{n} \biggr\} e^{\int^{t}_{t_{n}}(1-\alpha)p(\xi)\,d\xi} \\ &{}+(1-\alpha) \int^{t}_{t_{n}} q(s) e^{\int^{t}_{s}(1-\alpha)p(\xi)\,d\xi }\,ds \\ =& \biggl\{ v(t_{0})\prod_{t_{0}< t_{k}< t}E_{k}+ \sum_{t_{0}< t_{k}< t}\biggl[\prod_{t_{k}< t_{j}< t}E_{j} \biggr]G_{k} \biggr\} e^{\int^{t}_{t_{n}}(1-\alpha)p(\xi)\,d\xi} \\ &{}+(1-\alpha) \int^{t}_{t_{n}} q(s) e^{\int^{t}_{s}(1-\alpha)p(\xi)\,d\xi} \,ds. \end{aligned}$$

Hence (2.9) is valid on $[t_{n}, t_{n+1}]$. Therefore, the inequalities (2.9) is valid for $t_{0}\leq t\leq t_{n+1}$. We know that $v(t)=m^{1-\alpha}(t)$, this completes the proof of Theorem 2.1. □

If $d_{k}\equiv1$ in Theorem 2.1, we obtain the following theorem.

### Theorem 2.2

*Suppose that* (H_0_) *and* (H_1_) *hold*, $p, q\in C[\mathbb{R}_{+}, \mathbb{R}]$ *and for* $k=1, 2, \ldots$ , $t\geq t_{0}$,

2.15 $$\begin{aligned}& m'(t)\leq p(t)m(t)+q(t)m^{\alpha}(t),\quad t\neq t_{k}, \end{aligned}$$

2.16 $$\begin{aligned}& \Delta m^{1-\alpha}(t_{k})\leq\frac{c_{k}}{\Gamma(\beta_{k})} \int ^{t_{k}}_{t_{k-1}}(t_{k}-s)^{\beta_{k}-1} m^{1-\alpha}(s)\,ds+b_{k}, \end{aligned}$$

*where* $0<\alpha<1$, $c_{k}, d_{k}\geq0$, $\beta_{k}>0$ *and* $b_{k}$ ($k=1, 2, \ldots$) *are constants*, $\Delta m^{1-\alpha}(t_{k})=m^{1-\alpha }(t_{k}^{+})-m^{1-\alpha}(t_{k})$. *Then we have the following inequality*:

2.17 $$\begin{aligned} m(t)^{1-\alpha} \leq& \biggl\{ m^{1-\alpha}(t_{0})\prod _{t_{0}< t_{k}< t}M_{k}+\sum _{t_{0}< t_{k}< t} \biggl[\prod_{t_{k}< t_{j}< t}M_{j} \biggr]N_{k} \biggr\} e^{\int^{t}_{t_{l}}(1-\alpha)p(\xi)\,d\xi} \\ &{}+(1-\alpha) \int^{t}_{t_{0}} q(s) e^{\int^{t}_{s}(1-\alpha)p(\xi)\,d\xi}, \end{aligned}$$

*where*

$$\begin{aligned}& M_{k}=\frac{c_{k}}{\Gamma(\beta_{k})} \int ^{t_{k}}_{t_{k-1}}(t_{k}-s)^{\beta_{k}-1}e^{\int^{s}_{t_{k-1}}(1-\alpha )p(\xi)\,d\xi} \,ds+e^{\int^{t_{k}}_{t_{k-1}}(1-\alpha)p(\xi)\,d\xi}, \\& N_{k} = \frac{c_{k}}{\Gamma(\beta_{k})} \int^{t_{k}}_{t_{k-1}} \int ^{s}_{t_{k-1}}(t_{k}-s)^{\beta_{k}-1}(1- \alpha)q(\nu)e^{\int^{s}_{\nu }p(\xi)\,d\xi}\,d\nu \,ds+b_{k}. \end{aligned}$$

### Proof

As the proof of Theorem 2.1, from (2.11) we have

2.18 $$ m^{1-\alpha}(t)\leq m^{1-\alpha}(t_{0})e^{\int^{t}_{t_{0}}(1-\alpha )p(\xi)\,d\xi} +(1- \alpha) \int^{t}_{t_{0}}q(s) e^{\int^{t}_{s}(1-\alpha)p(\xi)\,d\xi} \,ds, $$

which means that (2.17) holds for $t\in[t_{0}, t_{1}]$.

Now we use the method of mathematical induction; suppose that (2.17) holds for $t\in[t_{0}, t_{n}]$, then

2.19 $$\begin{aligned} m^{1-\alpha}(t) \leq& \biggl\{ m^{1-\alpha}(t_{0})\prod _{t_{0}< t_{k}< t}M_{k}+\sum _{t_{0}< t_{k}< t} \biggl[\prod_{t_{k}< t_{j}< t}M_{j} \biggr]N_{k} \biggr\} e^{\int^{t_{n}}_{t_{n-1}}(1-\alpha)p(\xi)\,d\xi} \\ &{}+(1-\alpha) \int^{t}_{t_{0}} q(s) e^{\int^{t_{n}}_{s}(1-\alpha)p(\xi)\,d\xi} \,ds. \end{aligned}$$

Then, by (2.16),

$$\begin{aligned} m^{1-\alpha}\bigl(t_{n}^{+}\bigr) \leq& m^{1-\alpha}(t_{n})+\frac{c_{n}}{\Gamma (\beta_{n})} \int^{t_{n}}_{t_{n-1}}(t_{n}-s)^{\beta_{n}-1} m^{1-\alpha}(s)\,ds+b_{n} \\ \leq&\biggl\{ m^{1-\alpha}(t_{0})\prod _{t_{0}< t_{k}< t_{n}}M_{k}+\sum_{t_{0}< t_{k}< t_{n}} \biggl[\prod_{t_{k}< t_{j}< t_{n}}M_{j} \biggr]N_{k}\biggr\} e^{\int^{t_{n}}_{t_{n-1}}(1-\alpha)p(\xi)\,d\xi} \\ &{}+(1-\alpha) \int^{t_{n}}_{t_{0}} q(s) e^{\int^{t_{n}}_{s}(1-\alpha )p(\xi)\,d\xi}\,ds \\ &{}+\frac{c_{n}}{\Gamma(\beta_{n})} \int^{t_{n}}_{t_{n-1}}(t_{n}-s)^{\beta _{n}-1} \biggl\{ m^{1-\alpha}(t_{0})\prod_{t_{0}< t_{k}< s}M_{k}+ \sum_{t_{0}< t_{k}< s}\biggl[\prod_{t_{k}< t_{j}< s}M_{j} \biggr]N_{k}\biggr\} \\ &{}\times e^{\int^{s}_{t_{n-1}}(1-\alpha)p(\xi)\,d\xi}\,ds \\ &+\frac{c_{n}}{\Gamma(\beta_{n})} \int^{t_{n}}_{t_{n-1}} \int _{t_{n-1}}^{s}(t_{n}-s)^{\beta_{n}-1}(1- \alpha)q(\nu)e^{\int ^{t_{n}}_{\nu}(1-\alpha)p(\xi)\,d\xi} \,d\nu \,ds+b_{n} \\ =&\biggl\{ m^{1-\alpha}(t_{0})\prod _{t_{0}< t_{k}< t_{n}}M_{k}+\sum_{t_{0}< t_{k}< t_{n}} \biggl[\prod_{t_{k}< t_{j}< t_{n}}M_{j} \biggr]N_{k}\biggr\} \\ &{}\times\biggl[\frac{c_{n}}{\Gamma(\beta_{n})} \int ^{t_{n}}_{t_{n-1}}(t_{n}-s)^{\beta_{n}-1}e^{\int^{s}_{t_{n-1}}(1-\alpha )p(\xi)\,d\xi} \,ds+e^{\int^{t_{n}}_{t_{n-1}}(1-\alpha)p(\xi)\,d\xi}\biggr] \\ &{}+(1-\alpha) \int^{t_{n}}_{t_{0}} q(s) e^{\int^{t_{n}}_{s}(1-\alpha )p(\xi)\,d\xi} \,ds+N_{n} \\ =&\biggl\{ m^{1-\alpha}(t_{0})\prod _{t_{0}< t_{k}< t_{n}}M_{k}+\sum_{t_{0}< t_{k}< t_{n}} \biggl[\prod_{t_{k}< t_{j}< t_{n}}M_{j} \biggr]N_{k}\biggr\} M_{n} \\ &{}+(1-\alpha) \int^{t_{n}}_{t_{0}} q(s) e^{\int^{t_{n}}_{s}(1-\alpha )p(\xi)\,d\xi} \,ds+N_{n}. \end{aligned}$$

By Lemma 1.2, we have

$$\begin{aligned} m^{1-\alpha}\bigl(t_{n}^{+}\bigr) \leq& \biggl\{ m^{1-\alpha}(t_{0})\prod_{t_{0}< t_{k}< t_{n+1}}M_{k}+ \sum_{t_{0}< t_{k}< t_{n+1}}\biggl[\prod_{t_{k}< t_{j}< t_{n+1}}M_{j} \biggr]N_{k}\biggr\} \\ &{}+(1-\alpha) \int^{t_{n}}_{t_{0}} q(s) e^{\int^{t_{n}}_{s}(1-\alpha )p(\xi)\,d\xi}\,ds. \end{aligned}$$

For $t\in[t_{n}, t_{n+1}]$, (2.18) can be replaced by

$$\begin{aligned} m^{1-\alpha}(t) \leq& m^{1-\alpha}\bigl(t_{n}^{+} \bigr)e^{\int ^{t}_{t_{n}}(1-\alpha)p(\xi)\,d\xi} +(1-\alpha) \int^{t}_{t_{n}}q(s) e^{\int^{t}_{s}(1-\alpha)p(\xi)\,d\xi }\,ds \\ \leq&\biggl\{ \biggl\{ m^{1-\alpha}(t_{0})\prod _{t_{0}< t_{k}< t}M_{k}+\sum_{t_{0}< t_{k}< t} \biggl[\prod_{t_{k}< t_{j}< t}M_{j} \biggr]N_{k}\biggr\} \\ &{}+ (1-\alpha) \int^{t_{n}}_{t_{0}} q(s) e^{\int^{t_{n}}_{s}(1-\alpha )p(\xi)\,d\xi}\,ds \biggr\} e^{\int^{t}_{t_{n}}(1-\alpha)p(\xi)\,d\xi} \\ &{}+(1-\alpha) \int^{t}_{t_{n}}q(s) e^{\int^{t}_{s}(1-\alpha)p(\xi)\,d\xi }\,ds \\ =&\biggl\{ m^{1-\alpha}(t_{0})\prod _{t_{0}< t_{k}< t}M_{k}+\sum_{t_{0}< t_{k}< t} \biggl[\prod_{t_{k}< t_{j}< t}M_{j} \biggr]N_{k}\biggr\} e^{\int ^{t}_{t_{n}}(1-\alpha)p(\xi)\,d\xi} \\ &{}+(1-\alpha) \int^{t_{n}}_{t_{0}} q(s) e^{\int^{t_{n}}_{s}(1-\alpha )p(\xi)\,d\xi}dse^{\int^{t}_{t_{n}}(1-\alpha)p(\xi)\,d\xi} \\ &{}+(1-\alpha) \int^{t}_{t_{n}}q(s) e^{\int^{t}_{s}(1-\alpha)p(\xi)\,d\xi}\,ds. \end{aligned}$$

Therefore, the estimate (2.17) holds for $t\in[t_{0}, t_{n+1}]$. This completes the proof. □

Using different estimating methods, we have the following results.

### Theorem 2.3

*Suppose that all the hypotheses of Theorem* 2.1 *are fulfilled*. *Then*, *for* $t\geq{t_{0}}$, *we have*:

(i) *For* $k=1, 2,\ldots $ , *the following estimation holds*:

2.20 $$\begin{aligned} m(t) \leq& \biggl\{ \biggl\{ m^{1-\alpha}(t_{0})\prod _{t_{0}< t_{k}< t} \biggl[\frac {c_{k}}{\Gamma(\beta_{k})}\biggl(\frac{e^{\mu_{k}t_{k}}\Gamma(\beta _{k}^{2})}{\mu_{k}^{\beta_{k}^{2}}} \biggr)^{\frac{1}{\mu_{k}}} \biggl( \int^{t_{k}}_{t_{k-1}}e^{\nu_{k}(\int^{s}_{t_{k-1}}(1-\alpha)p(\xi)\,d\xi-s)}\,ds \biggr)^{\frac{1}{\nu_{k}}} \\ &{}+d_{k}e^{\int^{t_{k}}_{t_{k-1}}(1-\alpha)p(\xi)\,d\xi}\biggr] \\ &{}+\sum_{t_{0}< t_{k}< t}\biggl[\prod _{t_{k}< t_{j}< t} \biggl[\frac{c_{j}}{\Gamma (\beta_{j})}\biggl(\frac{e^{\mu_{j}t_{j}}\Gamma(\beta_{j}^{2})}{\mu_{j}^{\beta _{j}^{2}}} \biggr)^{\frac{1}{\mu_{j}}} \biggl( \int^{t_{j}}_{t_{j-1}}e^{\nu_{j}(\int^{s}_{t_{j-1}}(1-\alpha)p(\xi)\,d\xi-s)}\,ds \biggr)^{\frac{1}{\nu_{j}}} \\ &{}+d_{k}e^{\int^{t_{j}}_{t_{j-1}}(1-\alpha)p(\xi)\,d\xi}\biggr]\biggr]\biggl(\frac{c_{k}}{\Gamma(\beta_{k})}\biggl(\frac{e^{\mu_{k}t_{k}}\Gamma (\beta_{k}^{2})}{\mu_{k}^{\beta_{k}^{2}}}\biggr)^{\frac{1}{\mu_{k}}} \\ &{}\times \biggl( \int^{t_{k}} _{t_{k-1}}e^{-\nu_{k}s}\biggl\{ \int^{s}_{t_{k-1}}(1-\alpha)q(\nu )e^{\int^{s}_{\nu}(1-\alpha)p(\xi)\,d\xi}\,d \nu\biggr\} ^{\nu_{k}} \,ds\biggr)^{\frac {1}{\nu_{k}}} \\ &{}+d_{k} \int^{s}_{t_{k-1}}(1-\alpha)q(\nu)e^{\int^{s}_{\nu}(1-\alpha )p(\xi)\,d\xi}\,d \nu+b_{k} \biggr)\biggr\} e^{\int^{t}_{t_{l}}(1-\alpha)p(\xi)\,d\xi} \\ &{}+(1-\alpha) \int^{t} _{t_{l}} q(s)e^{\int^{t}_{s}(1-\alpha)p(\xi)\,d\xi }\,ds \biggr\} ^{\frac{1}{1-\alpha}}, \end{aligned}$$

*where* $\mu_{k}=\beta_{k}+1$ *and* $\nu_{k}=1+\frac{1}{\beta_{k}}$.

(ii) *If we assume further that* $\beta_{k}>\frac{1}{2}$, *then*, *for* $k=1, 2,\ldots $ , *we have the following estimation*:

2.21 $$\begin{aligned} m(t) \leq& \biggl\{ \biggl\{ m^{1-\alpha}(t_{0})\prod _{t_{0}\leq t_{k}\leq t} \biggl[\frac{c_{k}}{\Gamma(\beta_{k})}\biggl(\frac{e^{2t_{k}}\Gamma(2\beta _{k}-1)}{2^{2\beta_{k}-1}} \biggr)^{\frac{1}{2}} \biggl( \int^{t_{k}}_{t_{k-1}}e^{2(\int^{s}_{t_{k-1}}(1-\alpha)p(\xi)\,d\xi -s)}\,ds \biggr)^{\frac{1}{2}} \\ &{}+d_{k}e^{\int^{t_{k}}_{t_{k-1}}(1-\alpha)p(\xi)\,d\xi}\biggr] \\ &{}+\sum_{t_{0}< t_{k}< t}\biggl[\prod _{t_{k}< t_{j}< t} \biggl[\frac{c_{j}}{\Gamma (\beta_{j})}\biggl(\frac{e^{2t_{j}}\Gamma(2\beta_{j}-1)}{2^{2\beta _{j}-1}} \biggr)^{\frac{1}{2}} \biggl( \int^{t_{j}}_{t_{j-1}}e^{2(\int^{s}_{t_{j-1}}(1-\alpha)p(\xi)\,d\xi -s)}\,ds \biggr)^{\frac{1}{2}} \\ &{}+d_{k}e^{\int^{t_{j}}_{t_{j-1}}(1-\alpha)p(\xi)\,d\xi}\biggr]\biggr]\biggl(\frac{c_{k}}{\Gamma(\beta_{k})}\biggl(\frac{e^{2t_{k}}\Gamma(2\beta _{k}-1)}{2^{2\beta_{k}-1}}\biggr)^{\frac{1}{2}} \\ &{}\times \biggl( \int^{t_{k}} _{t_{k-1}}e^{-2s}\biggl\{ \int^{s}_{t_{k-1}}(1-\alpha)q(\nu )e^{\int^{s}_{\nu}(1-\alpha)p(\xi)\,d\xi}\,d \nu\biggr\} ^{2} \,ds\biggr)^{\frac {1}{2}} \\ &{}+d_{k} \int^{s}_{t_{k-1}}(1-\alpha)q(\nu)e^{\int^{s}_{\nu}(1-\alpha )p(\xi)\,d\xi}\,d \nu+b_{k} \biggr)\biggr\} e^{\int^{t}_{t_{l}}(1-\alpha)p(\xi)\,d\xi} \\ &{}+(1-\alpha) \int^{t} _{t_{l}} q(s)e^{\int^{t}_{s}(1-\alpha)p(\xi)\,d\xi }\,ds \biggr\} ^{\frac{1}{1-\alpha}}. \end{aligned}$$

### Proof

To prove (i), we use the Hölder inequality. For $k=1, 2,\ldots $ , we get

$$\begin{aligned}& \int^{t_{k}}_{t_{k-1}}(t_{k}-s)^{\beta_{k}-1}e^{\int ^{s}_{t_{k-1}}(1-\alpha)p(\xi)\,d\xi} \,ds \\ & \quad \leq\biggl( \int^{t_{k}}_{t_{k-1}}(t_{k}-s)^{(\beta_{k}-1)\mu_{k}}e^{\mu _{k}s} \,ds\biggr)^{\frac{1}{\mu_{k}}}\biggl( \int^{t_{k}}_{t_{k-1}}e^{-\nu_{k}s} e^{\nu_{k}\int_{t_{k-1}}^{s}(1-\alpha)p(\xi)\,d\xi}\,ds \biggr)^{\frac{1}{\nu _{k}}} \\ & \quad < \biggl(\frac{e^{\mu_{k}t_{k}}\Gamma(\beta_{k}^{2})}{\mu_{k}^{\beta _{k}^{2}}}\biggr)^{\frac{1}{\mu_{k}}} \biggl( \int^{t_{k}}_{t_{k-1}}e^{-\nu_{k}s}e^{\nu_{k}\int _{t_{k-1}}^{s}(1-\alpha)p(\xi)\,d\xi}\,ds \biggr)^{\frac{1}{\nu_{k}}}. \end{aligned}$$

In fact, by changing the variable, we get

$$\begin{aligned} \int^{t_{k}}_{t_{k-1}}(t_{k}-s)^{(\beta_{k}-1)\mu_{k}}e^{\mu_{k}s} \,ds =&e^{\mu_{k}t_{k}} \int_{0}^{t_{k}-t_{k-1}}\eta^{\mu_{k}(\beta _{k}-1)}e^{-\mu_{k}\eta} \,d\eta \\ =&\frac{e^{\mu_{k}t_{k}}}{\mu_{k}^{1-\mu_{k}(1-\beta_{k})}} \int _{0}^{\mu_{k}(t_{k}-t_{k-1})}\lambda^{\mu_{k}(\beta_{k}-1)}e^{-\lambda }d \lambda \\ < &\frac{e^{\mu_{k}t_{k}}}{\mu_{k}^{1-\mu_{k}(1-\beta_{k})}}\Gamma\bigl(1-\mu _{k}(1-\beta_{k}) \bigr) \\ =&\frac{e^{\mu_{k}t_{k}}\Gamma(\beta_{k}^{2})}{\mu_{k}^{\beta_{k}^{2}}} \end{aligned}$$

and

$$\begin{aligned}& \int^{t_{k}}_{t_{k-1}} \int^{s}_{t_{k-1}}(t_{k}-s)^{\beta_{k}-1}(1- \alpha )q(\nu)e^{\int^{s}_{\nu}(1-\alpha)p(\xi)\,d\xi}\,d\nu \,ds \\ & \quad \leq\biggl( \int^{t_{k}}_{t_{k-1}}(t_{k}-s)^{(\beta_{k}-1)\mu_{k}}e^{\mu _{k}s} \,ds\biggr)^{\frac{1}{\mu_{k}}} \\ & \qquad {}\times\biggl( \int^{t_{k}}_{t_{k-1}}e^{-\nu_{k}s} \biggl\{ \int_{t_{k-1}}^{s}(1-\alpha)q(\nu)e^{\int^{s}_{\nu}(1-\alpha)p(\xi)\,d\xi}\,d\nu \biggr\} ^{\nu_{k}}\,ds\biggr)^{\frac{1}{\nu_{k}}} \\ & \quad < \biggl(\frac{e^{\mu_{k}t_{k}}\Gamma(\beta_{k}^{2})}{\mu_{k}^{\beta _{k}^{2}}}\biggr)^{\frac{1}{\mu_{k}}} \biggl( \int^{t_{k}} _{t_{k-1}}e^{-\nu_{k}s}\biggl\{ \int^{s}_{t_{k-1}}(1-\alpha)q(\nu )e^{\int^{s}_{\nu}(1-\alpha)p(\xi)\,d\xi}\,d \nu\biggr\} ^{\nu_{k}} \,ds\biggr)^{\frac {1}{\nu_{k}}}. \end{aligned}$$

Substituting the above inequalities in (2.3), we obtain the desired inequality in (2.20).

To prove (ii), since in this case, $2\beta_{k}-1>0$, $\Gamma(2\beta_{k}-1)$ are well defined for $k=1, 2,\ldots $ . We use the Cauchy–Schwartz inequality to get

$$\begin{aligned}& \int^{t_{k}}_{t_{k-1}}(t_{k}-s)^{\beta_{k}-1}e^{\int ^{s}_{t_{k-1}}(1-\alpha)p(\xi)\,d\xi} \,ds \\& \quad \leq\biggl( \int^{t_{k}}_{t_{k-1}}(t_{k}-s)^{2(\beta_{k}-1)}e^{2s} \,ds\biggr)^{\frac {1}{2}}\biggl( \int^{t_{k}}_{t_{k-1}}e^{2(\int^{s}_{t_{k-1}}(1-\alpha)p(\xi)\,d\xi-s)}\,ds \biggr)^{\frac{1}{2}} \\& \quad < \biggl(\frac{e^{2t_{k}}\Gamma(2\beta_{k}-1)}{2^{2\beta_{k}-1}}\biggr)^{\frac{1}{2}} \biggl( \int^{t_{k}}_{t_{k-1}}e^{2(\int^{s}_{t_{k-1}}(1-\alpha)p(\xi)\,d\xi -s)}\,ds \biggr)^{\frac{1}{2}} \end{aligned}$$

and

$$\begin{aligned}& \int^{t_{k}}_{t_{k-1}} \int^{s}_{t_{k-1}}(t_{k}-s)^{\beta_{k}-1}(1- \alpha )q(\nu)e^{\int^{s}_{\nu}(1-\alpha)p(\xi)\,d\xi}\,d\nu \,ds \\& \quad \leq\biggl( \int^{t_{k}}_{t_{k-1}}(t_{k}-s)^{2(\beta_{k}-1)}e^{2s} \,ds\biggr)^{\frac {1}{2}}\biggl( \int^{t_{k}} _{t_{k-1}}e^{-2s}\biggl\{ \int^{s}_{t_{k-1}}(1-\alpha )q(\nu)e^{\int^{s}_{\nu}(1-\alpha)p(\xi)\,d\xi}\,d \nu\biggr\} ^{2} \,ds\biggr)^{\frac {1}{2}} \\& \quad < \biggl(\frac{e^{2t_{k}}\Gamma(2\beta_{k}-1)}{2^{2\beta_{k}-1}}\biggr)^{\frac{1}{2}} \biggl( \int^{t_{k}} _{t_{k-1}}e^{-2s}\biggl\{ \int^{s}_{t_{k-1}}(1-\alpha)q(\nu )e^{\int^{s}_{\nu}(1-\alpha)p(\xi)\,d\xi}\,d \nu\biggr\} ^{2} \,ds\biggr)^{\frac{1}{2}}. \end{aligned}$$

Substituting these two inequalities in (2.3), we get the desired results. The proof is completed. □

If $d_{k}\equiv0$ and $p(t)$ is constant function in Theorem 2.2, we obtain the following corollary.

### Corollary 2.4

*Suppose* (H_0_) *and* (H_1_) *hold*, *and for* $q\in C[\mathbb{R}_{+}, \mathbb{R}]$, $k=1, 2, \ldots$ , $t\geq t_{0}$,

2.22 $$\begin{aligned}& m'(t)\leq pm(t)+q(t)m^{\alpha}(t),\quad t\neq t_{k}, \end{aligned}$$

2.23 $$\begin{aligned}& m^{1-\alpha}\bigl(t_{k}^{+}\bigr)\leq\frac{c_{k}}{\Gamma(\beta_{k})} \int ^{t_{k}}_{t_{k-1}}(t_{k}-s)^{\beta_{k}-1} m^{1-\alpha}(s)\,ds+b_{k}, \end{aligned}$$

*where* $0<\alpha<1$, $c_{k}, d_{k}\geq0$, $\beta_{k}>0$ *and* $b_{k}$ ($k=1, 2, \ldots$) *are constants*. *Then*, *for* $t\geq t_{0}$, *we have the following estimates*.

*Case* I: $p\neq\frac{1}{1-\alpha}$:

(i) *For* $k=1, 2,\ldots $ , *the following estimation holds*:

2.24 $$\begin{aligned} m(t) \leq& \biggl\{ m^{1-\alpha}(t_{0}) \biggl(\prod _{t_{0}< t_{k}< t}A_{k}\biggr)e^{p(1-\alpha)(t-t_{0})}+\sum _{t_{0}< t_{k}< t}\biggl(\prod_{t_{k}< t_{j}< t} A_{j}\biggr)B_{k}e^{p(1-\alpha)(t-t_{k})} \\ &{}+ \int^{t}_{t_{l}}(1-\alpha)q(s)e^{p(1-\alpha)(t-s)}\,ds \biggr\} ^{\frac {1}{1-\alpha}}, \end{aligned}$$

*where*

$$\begin{aligned}& A_{k}=\frac{c_{k}}{\Gamma(\beta_{k})}\biggl(\frac{\Gamma(\beta_{k}^{2})}{\mu _{k}^{\beta_{k}^{2}}} \biggr)^{\frac{1}{\mu_{k}}} \biggl(\frac{1-e^{\nu_{k}(t_{k}-t_{k-1})(1-(1-\alpha)p)}}{\nu_{k}((1-\alpha )p-1)}\biggr)^{\frac{1}{\nu_{k}}}, \\& \begin{aligned} B_{k}&=\frac{c_{k}}{\Gamma(\beta_{k})}\biggl(\frac{e^{\mu_{k}t_{k}}\Gamma(\beta _{k}^{2})}{\mu_{k}^{\beta_{k}^{2}}} \biggr)^{\frac{1}{\mu_{k}}} \biggl( \int^{t_{k}} _{t_{k-1}}e^{\nu_{k}((1-\alpha)p-1)s}\biggl\{ \int ^{s}_{t_{k-1}}(1-\alpha)q(\nu)e^{-(1-\alpha)p\nu}\,d \nu\biggr\} ^{\nu_{k}} \,ds\biggr)^{\frac{1}{\nu_{k}}} \\ &\quad {}+b_{k}. \end{aligned} \end{aligned}$$

(ii) *If we assume further* $\beta_{k}>\frac{1}{2}$, *then*, *for* $k=1, 2, \ldots $ ,

2.25 $$\begin{aligned} m(t) \leq& \biggl\{ m^{1-\alpha}(t_{0}) \biggl(\prod _{t_{0}< t_{k}< t}C_{k}\biggr)e^{p(1-\alpha)(t-t_{0})}+\sum _{t_{0}< t_{k}< t}\biggl(\prod_{t_{k}< t_{j}< t} C_{j}\biggr)D_{k}e^{p(1-\alpha)(t-t_{k})} \\ &{}+ \int^{t}_{t_{l}}(1-\alpha)q(s)e^{p(1-\alpha)(t-s)}\,ds \biggr\} ^{\frac {1}{1-\alpha}}, \end{aligned}$$

*where*

$$\begin{aligned}& C_{k}=\frac{c_{k}}{2^{\beta_{k}}\Gamma(\beta_{k})}\biggl\{ \frac{\Gamma(2\beta _{k}-1)}{(1-\alpha)p-1} \bigl[1-e^{2(t_{k}-t_{k-1})(1-(1-\alpha)p)}\bigr]\biggr\} ^{\frac{1}{2}}, \\& \begin{aligned} D_{k}&=\frac{c_{k}}{\Gamma(\beta_{k})}\biggl(\frac{e^{2t_{k}}\Gamma(2\beta _{k}-1)}{2^{2\beta_{k}-1}} \biggr)^{\frac{1}{2}} \biggl( \int^{t_{k}} _{t_{k-1}}e^{2((1-\alpha)p-1)s}\biggl\{ \int ^{s}_{t_{k-1}}(1-\alpha)q(\nu)e^{-(1-\alpha)p\nu}\,d \nu\biggr\} ^{2} \,ds\biggr)^{\frac {1}{2}} \\ &\quad {}+b_{k}. \end{aligned} \end{aligned}$$

*Case* II: $p=\frac{1}{1-\alpha}$:

(i) *For* $k=1, 2,\ldots $ , *the following estimation holds*:

2.26 $$\begin{aligned} m(t) \leq& \biggl\{ m^{1-\alpha}(t_{0}) \biggl(\prod _{t_{0}< t_{k}< t}E_{k}\biggr)e^{(t-t_{0})}+\sum _{t_{0}< t_{k}< t}\biggl(\prod_{t_{k}< t_{j}< t} E_{j}\biggr)F_{k}e^{(t-t_{k})} \\ &{}+ \int^{t}_{t_{l}}(1-\alpha)q(s)e^{(t-s)}\,ds \biggr\} ^{\frac{1}{1-\alpha}}, \end{aligned}$$

*where*

$$\begin{aligned}& E_{k}=\frac{c_{k}}{\Gamma(\beta_{k})}\biggl(\frac{\Gamma(\beta_{k}^{2})}{\mu _{k}^{\beta_{k}^{2}}} \biggr)^{\frac{1}{\mu_{k}}} (t_{k}-t_{k-1})^{\frac{1}{\nu_{k}}}, \\& F_{k}=\frac{c_{k}}{\Gamma(\beta_{k})}\biggl(\frac{e^{\mu_{k}t_{k}}\Gamma(\beta _{k}^{2})}{\mu_{k}^{\beta_{k}^{2}}} \biggr)^{\frac{1}{\mu_{k}}} \biggl( \int^{t_{k}} _{t_{k-1}}\biggl\{ \int^{s}_{t_{k-1}}(1-\alpha)q(\nu)e^{-\nu }\,d \nu\biggr\} ^{\nu_{k}} \,ds\biggr)^{\frac{1}{\nu_{k}}}+b_{k}. \end{aligned}$$

(ii) *If we assume further* $\beta_{k}>\frac{1}{2}$, *then*, *for* $k=1, 2, \ldots $ ,

2.27 $$\begin{aligned} m(t) \leq& \biggl\{ m^{1-\alpha}(t_{0}) \biggl(\prod _{t_{0}< t_{k}< t}G_{k}\biggr)e^{(t-t_{0})}+\sum _{t_{0}< t_{k}< t}\biggl(\prod_{t_{k}< t_{j}< t} G_{j}\biggr)H_{k}e^{(t-t_{k})} \\ &{}+ \int^{t}_{t_{l}}(1-\alpha)q(s)e^{(t-s)}\,ds \biggr\} ^{\frac{1}{1-\alpha}}, \end{aligned}$$

*where*

$$\begin{aligned}& G_{k}=\frac{c_{k}}{\Gamma(\beta_{k})}\biggl(\frac{\Gamma(2\beta _{k}-1)(t_{k}-t_{k-1})}{2^{2\beta_{k}-1}} \biggr)^{\frac{1}{2}}, \\& H_{k}=\frac{c_{k}}{\Gamma(\beta_{k})}\biggl(\frac{e^{2t_{k}}\Gamma(2\beta _{k}-1)}{2^{2\beta_{k}-1}} \biggr)^{\frac{1}{2}} \biggl( \int^{t_{k}} _{t_{k-1}}\biggl\{ \int^{s}_{t_{k-1}}(1-\alpha)q(\nu)e^{-\nu }\,d \nu\biggr\} ^{2}\,ds\biggr)^{\frac{1}{2}}+b_{k}. \end{aligned}$$

If $p(t)\equiv0$ and $d_{k}\equiv1$ in Theorem 2.3, we obtain the following corollary.

### Corollary 2.5

*Let* (H_0_) *and* (H_1_) *hold*, *and for* $q\in C[\mathbb{R}_{+}, \mathbb{R}]$, $k=1, 2, \ldots$ , $t\geq t_{0}$

2.28 $$\begin{aligned}& m'(t)\leq q(t)m^{\alpha}(t),\quad t\neq t_{k}, \end{aligned}$$

2.29 $$\begin{aligned}& \Delta m^{1-\alpha}\bigl(t_{k}^{+}\bigr)\leq \frac{c_{k}}{\Gamma(\beta_{k})} \int ^{t_{k}}_{t_{k-1}}(t_{k}-s)^{\beta_{k}-1} m^{1-\alpha}(s)\,ds+b_{k}, \end{aligned}$$

*where* $0<\alpha<1$, $c_{k}, d_{k}\geq0$, $\beta_{k}>0$ *and* $b_{k}$ ($k=1, 2, \ldots$) *are constants*, $\Delta m^{1-\alpha}(t_{k})=m^{1-\alpha }(t_{k}^{+})-m^{1-\alpha}(t_{k})$. *Then*, *for* $t\geq t_{0}$, *we have*:

(i) *For* $k=1, 2,\ldots $ , *the following estimation holds*:

2.30 $$\begin{aligned} m(t) \leq& \biggl\{ m^{1-\alpha}(t_{0}) \biggl(\prod _{t_{0}< t_{k}< t}I_{k}\biggr)+\sum _{t_{0}< t_{k}< t}\biggl(\prod_{t_{k}< t_{j}< t} I_{j}\biggr)L_{k} \\ &{}+ \int^{t}_{t_{l}}(1-\alpha)q(s)\,ds\biggr\} ^{\frac{1}{1-\alpha}}, \end{aligned}$$

*where*

$$\begin{aligned}& I_{k}=1+\frac{c_{k}}{\Gamma(\beta_{k})}\biggl(\frac{\Gamma(\beta_{k}^{2})}{\mu _{k}^{\beta_{k}^{2}}} \biggr)^{\frac{1}{\mu_{k}}} \biggl(\frac{e^{\nu_{k}(t_{k}-t_{k-1})-1}}{\nu_{k}}\biggr)^{\frac{1}{\nu_{k}}}, \\& \begin{aligned} L_{k}&= \int^{t_{k}}_{t_{k-1}}q(s)\,ds+\frac{c_{k}}{\Gamma(\beta _{k})}\biggl( \frac{e^{\mu_{k}t_{k}}\Gamma(\beta_{k}^{2})}{\mu_{k}^{\beta _{k}^{2}}}\biggr)^{\frac{1}{\mu_{k}}} \\ &\quad {}\times\biggl( \int^{t_{k}} _{t_{k-1}}e^{-\nu_{k}s}\biggl\{ \int^{s}_{t_{k-1}}(1-\alpha)q(\nu )e^{-(1-\alpha)p\nu}\,d \nu\biggr\} ^{\nu_{k}} \,ds\biggr)^{\frac{1}{\nu_{k}}} \\ &\quad {}+b_{k}. \end{aligned} \end{aligned}$$

(ii) *If we assume further* $\beta_{k}>\frac{1}{2}$, *then*, *for* $k=1, 2, \ldots $ ,

2.31 $$\begin{aligned} m(t) \leq& \biggl\{ m^{1-\alpha}(t_{0}) \biggl(\prod _{t_{0}< t_{k}< t}M_{k}\biggr)+\sum _{t_{0}< t_{k}< t}\biggl(\prod_{t_{k}< t_{j}< t} M_{j}\biggr)N_{k} \\ &{}+ \int^{t}_{t_{l}}(1-\alpha)q(s)\,ds\biggr\} ^{\frac{1}{1-\alpha}}, \end{aligned}$$

*where*

$$\begin{aligned}& M_{k}=1+\frac{c_{k}}{2^{\beta_{k}}\Gamma(\beta_{k})}\biggl\{ \frac{\Gamma (2\beta_{k}-1)}{2^{2\beta_{k}}} \bigl[e^{2(t_{k}-t_{k-1})}\bigr]\biggr\} ^{\frac{1}{2}}, \\& \begin{aligned} N_{k}&= \int^{t_{k}}_{t_{k-1}}q(s)\,ds+\frac{c_{k}}{\Gamma(\beta_{k})}\biggl( \frac {e^{2t_{k}}\Gamma(2\beta_{k}-1)}{2^{2\beta_{k}-1}}\biggr)^{\frac{1}{2}} \biggl( \int^{t_{k}} _{t_{k-1}}e^{-2s}\biggl\{ \int^{s}_{t_{k-1}}(1-\alpha)q(\nu)\,d\nu \biggr\} ^{2} \,ds\biggr)^{\frac{1}{2}} \\ &\quad {}+b_{k}. \end{aligned} \end{aligned}$$

Next, we give another kind of nonlinear impulsive differential inequalities.

### Theorem 2.6

*Suppose that* (H_0_) *and* (H_1_) *hold*, $p, q\in C[\mathbb{R}_{+}, \mathbb{R}]$ *and for* $k=1, 2, \ldots$ , $t\geq t_{0}$,

2.32 $$\begin{aligned}& m'(t)\leq p(t)m(t)+q(t)m^{\alpha}(t),\quad t\neq t_{k}, \end{aligned}$$

2.33 $$\begin{aligned}& \Delta m(t_{k})\leq\frac{c_{k}}{\Gamma(\beta_{k})} \int ^{t_{k}}_{t_{k-1}}(t_{k}-s)^{\beta_{k}-1} m(s)\,ds+b_{k}, \end{aligned}$$

*where* $0<\alpha<1$, $c_{k}, d_{k}\geq0$, $\beta_{k}>0$ *and* $b_{k}$ ($k=1, 2, \ldots$) *are constants*, $\Delta m(t_{k})=m(t_{k}^{+})-m(t_{k})$. *Then we have the following estimation*:

2.34 $$\begin{aligned} m(t) \leq& \biggl\{ \biggl\{ m(t_{0})\prod _{t_{0}< t_{k}< t}P_{k}+\sum_{t_{0}< t_{k}< t} \biggl[\prod_{t_{k}< t_{j}< t}P_{j} \biggr]Q_{k}\biggr\} ^{1-\alpha}e^{\int ^{t}_{t_{l}}(1-\alpha)p(\xi)\,d\xi} \\ &{}+(1-\alpha)2^{(k-1)\alpha} \int_{t_{0}}^{t}q(s) e^{\int ^{t}_{s}(1-\alpha)p(\xi)\,d\xi}\,ds\biggr\} ^{\frac{1}{1-\alpha}}, \quad t\geq t_{0}, \end{aligned}$$

*where*

$$\begin{aligned}& P_{k}=2^{\frac{\alpha}{1-\alpha}}\biggl[e^{\int ^{t_{k}}_{t_{k-1}}p(\xi)\,d\xi}+ \frac{c_{k}}{\Gamma(\beta_{k})} \int^{t_{k}}_{t_{k-1}}(t_{k}-s)^{\beta _{k}-1}e^{\int^{s}_{t_{k-1}}p(\xi)\,d\xi} \,ds \biggr], \\& Q_{k}=\frac{c_{k}}{\Gamma(\beta_{k})}2^{\frac{(k-1)\alpha}{1-\alpha }}(1-\alpha)^{\frac{1}{1-\alpha}} \int^{t_{k}}_{t_{k-1}} \biggl( \int^{s}_{t_{0}}q(\nu)e^{\int^{s}_{\nu}(1-\alpha)p(\xi)\,d\xi}\,d\nu \biggr)^{\frac{1}{1-\alpha}}+b_{k}. \end{aligned}$$

### Proof

Obviously, using (2.11), we have (2.34) holds for $t\in [t_{0}, t_{1}]$. Suppose (2.34) holds for $t\in[t_{0}, t_{n}]$, then, by mathematical induction, we see that

$$\begin{aligned} m^{1-\alpha}(t_{n}) \leq& \biggl\{ \biggl\{ m(t_{0}) \prod_{t_{0}< t_{k}< t_{n}}P_{k}+\sum _{t_{0}< t_{k}< t_{n}}\biggl[\prod_{t_{k}< t_{j}< t_{n}}P_{j} \biggr]Q_{k}\biggr\} ^{1-\alpha}e^{\int^{t_{n}}_{t_{n-1}}(1-\alpha)p(\xi )\,d\xi} \\ &{}+(1-\alpha)2^{(n-1)\alpha} \int_{t_{0}}^{t_{n}}q(s) e^{\int ^{t_{n}}_{s}(1-\alpha)p(\xi)\,d\xi}\,ds\biggr\} . \end{aligned}$$

Since $\frac{1}{1-\alpha}>1$, by Lemma 1.1,

$$\begin{aligned} m(t_{n}) \leq& 2^{\frac{\alpha}{1-\alpha}}\biggl\{ m(t_{0})\prod _{t_{0}< t_{k}< t_{n}}P_{k}+\sum _{t_{0}< t_{k}< t_{n}}\biggl[\prod_{t_{k}< t_{j}< t_{n}}P_{j} \biggr]Q_{k}\biggr\} e^{\int^{t_{n}}_{t_{n-1}}p(\xi)\,d\xi} \\ &{}+2^{\frac{\alpha}{1-\alpha}}2^{\frac{(n-1)\alpha}{1-\alpha}}(1-\alpha )^{\frac{1}{1-\alpha}}\biggl( \int_{t_{0}}^{t_{n}}q(s) e^{\int ^{t_{n}}_{s}(1-\alpha)p(\xi)\,d\xi}\,ds \biggr)^{\frac{1}{1-\alpha}} \\ =& 2^{\frac{\alpha}{1-\alpha}}\biggl\{ m(t_{0})\prod _{t_{0}< t_{k}< t_{n}}P_{k}+\sum_{t_{0}< t_{k}< t_{n}} \biggl[\prod_{t_{k}< t_{j}< t_{n}}P_{j} \biggr]Q_{k}\biggr\} e^{\int^{t_{n}}_{t_{n-1}}p(\xi)\,d\xi} \\ &{}+2^{\frac{n\alpha}{1-\alpha}}(1-\alpha)^{\frac{1}{1-\alpha}}\biggl( \int _{t_{0}}^{t_{n}}q(s) e^{\int^{t_{n}}_{s}(1-\alpha)p(\xi)\,d\xi}\,ds \biggr)^{\frac {1}{1-\alpha}}, \end{aligned}$$

thus

$$\begin{aligned} m\bigl(t_{n}^{+}\bigr) \leq& m(t_{n})+ \frac{c_{n}}{\Gamma(\beta_{n})} \int ^{t_{n}}_{t_{n-1}}(t_{n}-s)^{\beta_{n}-1} m(s)\,ds+b_{n} \\ \leq& 2^{\frac{\alpha}{1-\alpha}}\biggl\{ m(t_{0})\prod _{t_{0}< t_{k}< t_{n}}P_{k}+\sum_{t_{0}< t_{k}< t_{n}} \biggl[\prod_{t_{k}< t_{j}< t_{n}}P_{j} \biggr]Q_{k}\biggr\} e^{\int^{t_{n}}_{t_{n-1}}p(\xi)\,d\xi} \\ &{}+2^{\frac{n\alpha}{1-\alpha}}(1-\alpha)^{\frac{1}{1-\alpha}}\biggl( \int _{t_{0}}^{t_{n}}q(s) e^{\int^{t_{n}}_{s}(1-\alpha)p(\xi)\,d\xi}\,ds \biggr)^{\frac {1}{1-\alpha}} \\ &{}+\frac{c_{n}}{\Gamma(\beta_{n})}2^{\frac{\alpha}{1-\alpha}} \int ^{t_{n}}_{t_{n-1}}(t_{n}-s)^{\beta_{n}-1} \biggl\{ m(t_{0})\prod_{t_{0}< t_{k}< t_{n}}P_{k}+ \sum_{t_{0}< t_{k}< t_{n}}\biggl[\prod_{t_{k}< t_{j}< t_{n}}P_{j} \biggr]Q_{k}\biggr\} \\ &{}\times e^{\int^{t_{n}}_{t_{n-1}}p(\xi)\,d\xi}\,ds \\ &{}+\frac{c_{n}}{\Gamma(\beta_{n})}2^{\frac{n\alpha}{1-\alpha}}(1-\alpha )^{\frac{1}{1-\alpha}} \int^{t_{n}}_{t_{n-1}}(t_{n}-s)^{\beta_{n}-1} \biggl( \int_{t_{0}}^{t_{n}}q(s)e^{\int^{t_{n}}_{s}(1-\alpha)p(\xi)\,d\xi }\,ds \biggr)^{\frac{1}{1-\alpha}}\,d\nu \\ =& \biggl\{ m(t_{0})\prod_{t_{0}< t_{k}< t_{n}}P_{k}+ \sum_{t_{0}< t_{k}< t_{n}}\biggl[\prod_{t_{k}< t_{j}< t_{n}}P_{j} \biggr]Q_{k}\biggr\} \\ &{}\times\biggl[2^{\frac{\alpha}{1-\alpha}}\biggl(e^{\int^{t_{n}}_{t_{n-1}}p(\xi)\,d\xi}+\frac{c_{n}}{\Gamma(\beta_{n})} \int ^{t_{n}}_{t_{n-1}}(t_{n}-s)^{\beta_{n}-1}e^{\int^{t_{n}}_{s}(1-\alpha )p(\xi)\,d\xi} \,ds\biggr)\biggr]+R_{n} \\ &{}+2^{\frac{n\alpha}{1-\alpha}}(1-\alpha)^{\frac{1}{1-\alpha}}\biggl( \int _{t_{0}}^{t_{n}}q(s)e^{\int^{t_{n}}_{s}(1-\alpha)p(\xi)\,d\xi}\,ds \biggr)^{\frac {1}{1-\alpha}} \\ =&\biggl\{ m(t_{0})\prod_{t_{0}< t_{k}< t_{n}}P_{k}+ \sum_{t_{0}< t_{k}< t_{n}}\biggl[\prod_{t_{k}< t_{j}< t_{n}}P_{j} \biggr]Q_{k}\biggr\} P_{n}+R_{n} \\ &{}+2^{\frac{n\alpha}{1-\alpha}}(1-\alpha)^{\frac{1}{1-\alpha}}\biggl( \int _{t_{0}}^{t_{n}}q(s)e^{\int^{t_{n}}_{s}(1-\alpha)p(\xi)\,d\xi}\,ds \biggr)^{\frac {1}{1-\alpha}} \\ =&\biggl\{ m(t_{0})\prod_{t_{0}< t_{k}< t_{n+1}}P_{k}+ \sum_{t_{0}< t_{k}< t_{n+1}}\biggl[\prod_{t_{k}< t_{j}< t_{n+1}}P_{j} \biggr]Q_{k}\biggr\} \\ &{}+2^{\frac{n\alpha}{1-\alpha}}(1-\alpha)^{\frac{1}{1-\alpha}}\biggl( \int _{t_{0}}^{t_{n}}q(s)e^{\int^{t_{n}}_{s}(1-\alpha)p(\xi)\,d\xi}\,ds \biggr)^{\frac {1}{1-\alpha}}. \end{aligned}$$

Then, for $t\in[t_{n}, t_{n+1}]$, since $0<1-\alpha<1$, by Lemma 1.1 and (2.11), we obtain

$$\begin{aligned} m^{1-\alpha}(t) \leq& m^{1-\alpha}\bigl(t_{n}^{+} \bigr)e^{\int ^{t}_{t_{n}}(1-\alpha)p(\xi)\,d\xi} +(1-\alpha) \int^{t}_{t_{n}}q(s) e^{\int^{t}_{s}(1-\alpha)p(\xi)\,d\xi }\,ds \\ \leq& \biggl\{ \biggl\{ m(t_{0})\prod_{t_{0}< t_{k}< t}P_{k}+ \sum_{t_{0}< t_{k}< t}\biggl[\prod_{t_{k}< t_{j}< t}P_{j} \biggr]Q_{k}\biggr\} +2^{\frac{n\alpha}{1-\alpha }}(1-\alpha)^{\frac{1}{1-\alpha}} \\ &{}\times\biggl( \int_{t_{0}}^{t_{n}}q(s)e^{\int^{t_{n}}_{s}(1-\alpha)p(\xi)\,d\xi}\,ds \biggr)^{\frac{1}{1-\alpha}}\biggr\} ^{1-\alpha} \\ &{}\times e^{\int^{t}_{t_{n}}(1-\alpha)p(\xi)\,d\xi} +(1-\alpha) \int^{t}_{t_{n}}q(s) e^{\int^{t}_{s}(1-\alpha)p(\xi)\,d\xi }\,ds \\ \leq& \biggl\{ \biggl\{ m(t_{0})\prod_{t_{0}< t_{k}< t}P_{k}+ \sum_{t_{0}< t_{k}< t}\biggl[\prod_{t_{k}< t_{j}< t}P_{j} \biggr]Q_{k}\biggr\} ^{1-\alpha} \\ &{}+2^{n\alpha}(1-\alpha) \int_{t_{0}}^{t_{n}}q(s)e^{\int ^{t_{n}}_{s}(1-\alpha)p(\xi)\,d\xi}\,ds\biggr\} \\ &{}\times e^{\int^{t}_{t_{n}}(1-\alpha)p(\xi)\,d\xi} +(1-\alpha) \int^{t}_{t_{n}}q(s) e^{\int^{t}_{s}(1-\alpha)p(\xi)\,d\xi }\,ds \\ \leq&\biggl\{ m(t_{0})\prod_{t_{0}< t_{k}< t}P_{k}+ \sum_{t_{0}< t_{k}< t}\biggl[\prod_{t_{k}< t_{j}< t}P_{j} \biggr]Q_{k}\biggr\} ^{1-\alpha}e^{\int ^{t}_{t_{n}}(1-\alpha)p(\xi)\,d\xi} \\ &{}+2^{n\alpha}(1-\alpha) \int_{t_{0}}^{t}q(s)e^{\int^{t}_{s}(1-\alpha )p(\xi)\,d\xi}\,ds. \end{aligned}$$

Therefore, the estimation (2.34) is valid on $[t_{n}, t_{n+1}]$. This completes the proof. □

Now, we present and prove a bound for the solutions of nonlinear impulsive integral inequalities with nonlocal jump conditions.

### Theorem 2.7

*Assume that* (H_0_) *and* (H_1_) *hold*, $p, q, m \in C[\mathbb{R}_{+}, \mathbb{R}_{+}]$, *and*, *for* $k=1, 2, \ldots$ , $t\geq t_{0}$,

2.35 $$\begin{aligned} m(t) \leq& c+ \int^{t}_{t_{0}}p(s)m(s)\,ds+ \int^{t}_{t_{0}}q(s)m^{\alpha }(s)\,ds \\ &{}+\sum _{t_{0}< t_{k}< t}\frac{\gamma_{k}}{\Gamma(\beta_{k})} \int ^{t_{k}}_{t_{k-1}}(t_{k}-s)^{\beta_{k}-1} m(s)\,ds, \end{aligned}$$

*where* $0<\alpha<1$, $\gamma_{k}\geq0$, $\beta_{k}>0$ ($k=1, 2, \ldots$) *and* *c* *are constants*. *Then*, *for* $t\geq t_{0}$, *the following assertions hold*:

2.36 $$\begin{aligned} m(t) \leq& \biggl\{ \biggl\{ c\prod_{t_{0}< t_{k}< t}R_{k}+ \sum_{t_{0}< t_{k}< t} \biggl[\prod_{t_{k}< t_{j}< t}R_{j} \biggr]S_{k} \biggr\} ^{1-\alpha}e^{\int^{t}_{t_{l}}(1-\alpha)p(\xi)\,d\xi} \\ &{}+(1-\alpha)2^{(k-1)\alpha} \int_{t_{0}}^{t}q(s) e^{\int ^{t}_{s}(1-\alpha)p(\xi)\,d\xi}\,ds\biggr\} ^{\frac{1}{1-\alpha}}, \end{aligned}$$

*where*

$$\begin{aligned}& R_{k}=2^{\frac{\alpha}{1-\alpha}}\biggl[e^{\int^{t_{k}}_{t_{k-1}}p(\xi)\,d\xi }+\frac{\gamma_{k}}{\Gamma(\beta_{k})} \int ^{t_{k}}_{t_{k-1}}(t_{k}-s)^{\beta_{k}-1}e^{\int^{t_{k}}_{s}p(\xi)\,d\xi } \,ds \biggr], \\& S_{k}=\frac{\gamma_{k}}{\Gamma(\beta_{k})}2^{\frac{(k-1)\alpha}{1-\alpha }}(1-\alpha)^{\frac{1}{1-\alpha}} \int^{t_{k}}_{t_{k-1}} \biggl( \int^{s}_{t_{0}}q(\nu)e^{\int^{s}_{\nu}(1-\alpha)p(\xi)\,d\xi}\,d\nu \biggr)^{\frac{1}{1-\alpha}}+b_{k}. \end{aligned}$$

### Proof

We denote by $g(t)$ the right-side function of (2.35), and $g(t_{0})=c$. Then we get

$$\textstyle\begin{cases} g'(t)= p(t)m(t)+q(t)m^{\alpha}(t),\quad t\neq t_{k}, \\ g(t_{k}^{+})=\frac{\gamma_{k}}{\Gamma(\beta_{k})}\int ^{t_{k}}_{t_{k-1}}(t_{k}-s)^{\beta_{k}-1}m(s)\,ds+g(t_{k}), \end{cases} $$

Since $m(t)\leq g(t)$, we have

$$\textstyle\begin{cases} g'(t)\leq p(t)g(t)+q(t)m^{\alpha}(t), \quad t\neq t_{k}, \\ g(t_{k}^{+})\leq\frac{\gamma_{k}}{\Gamma(\beta_{k})}\int ^{t_{k}}_{t_{k-1}}(t_{k}-s)^{\beta_{k}-1}g(s)\,ds+g(t_{k}). \end{cases} $$

Applying Theorem 2.6, we deduce the estimation of $g(t)$ as

2.37 $$\begin{aligned} g(t) \leq& \biggl\{ \biggl\{ c\prod_{t_{0}< t_{k}< t}R_{k}+ \sum_{t_{0}< t_{k}< t}\biggl[\prod_{t_{k}< t_{j}< t}R_{j} \biggr]S_{k}\biggr\} ^{1-\alpha}e^{\int ^{t}_{t_{l}}(1-\alpha)p(\xi)\,d\xi} \\ &{}+(1-\alpha)2^{(k-1)\alpha} \int_{t_{0}}^{t}q(s) e^{\int ^{t}_{s}(1-\alpha)p(\xi)\,d\xi}\,ds\biggr\} ^{\frac{1}{1-\alpha}}. \end{aligned}$$

Moreover, $m(t)\leq g(t)$, this completes the proof. □

## Impulsive fractional differential and integral equations with integral jump conditions

In this section, we give some examples about impulsive nonlinear differential and integral inequalities with Riemann–Liouville fractional integral jump conditions.

### Definition 3.1

The Riemann–Liouville fractional integral of order $\alpha>0$ of a function *f*: $[t_{0}, \infty)\rightarrow\mathbb{R}$ is defined by

$$\bigl(I_{t_{0}}^{\alpha}f\bigr) (t)=\frac{1}{\Gamma(\alpha)} \int _{t_{0}}^{t}(t-x)^{\alpha-1}f(x)\,dx, $$

where $\Gamma(\cdot)$ is the Gamma function.

### Proposition 3.2

*Suppose that* $y\in PC^{1}[J, \mathbb{R}]$ *which satisfies*

$$\textstyle\begin{cases} y'(t)- Ry(t)+a(t)y^{\alpha}(t)\leq0, \quad t\neq t_{k}, t\in J=[0, T], \\ y^{1-\alpha}(t_{k}^{+})\leq c_{k}(I^{\beta_{k}}_{t_{k-1}}y^{1-\alpha })(t_{k})-b_{k}, \quad k=1, 2, \ldots, n, \\ y^{1-\alpha}(0)=y^{1-\alpha}(T)+\theta, \end{cases} $$

*where* $R>0$, $a\in C[\mathbb{R}_{+}, \mathbb{R}_{+}]$, $0=t_{0}< t_{1}< t_{2}<\cdots<t_{n}<t_{n+1}=T$, $0<\alpha<1$, $c_{k}, b_{k}\geq0$, $\beta_{k}>0$ ($k=1, 2, \ldots, n$) *and* *θ* *are constants*. *If either of the following four cases fulfilled*:

(i) $R\neq\frac{1}{1-\alpha}$ *and for* $k=1, 2,\ldots, n$, *the following hypotheses hold*:

$$\begin{aligned}& (\mathrm{P}_{1})\quad \prod_{k=1}^{n} \frac{c_{k}}{\Gamma(\beta_{k})}\biggl(\frac{\Gamma(\beta _{k}^{2})}{\mu_{k}^{\beta_{k}^{2}}}\biggr)^{\frac{1}{\mu_{k}}} \biggl( \frac{1-e^{\nu_{k}(t_{k}-t_{k-1})(1-(1-\alpha)p)}}{\nu_{k}((1-\alpha )p-1)}\biggr)^{\frac{1}{\nu_{k}}}< e^{-(1-\alpha)RT}, \\& (\mathrm{P}_{2})\quad \frac{c_{k}}{\Gamma(\beta_{k})}\biggl(\frac{e^{\mu_{k}t_{k}}\Gamma(\beta _{k}^{2})}{\mu_{k}^{\beta_{k}^{2}}} \biggr)^{\frac{1}{\mu_{k}}} \\& \hphantom{(\mathrm{P}_{2})\quad}\quad {}\times\biggl( \int^{t_{k}} _{t_{k-1}}e^{\nu_{k}((1-\alpha)R-1)s}\biggl\{ - \int ^{s}_{t_{k-1}}(1-\alpha)a(\nu)e^{-(1-\alpha)R\nu}\,d \nu\biggr\} ^{\nu_{k}} \,ds\biggr)^{\frac{1}{\nu_{k}}}\leq b_{k}, \\& (\mathrm{P}_{3})\quad \theta\leq(1-\alpha) \int_{t_{n}}^{t}a(s)e^{(1-\alpha)R(T-s)}\,ds, \end{aligned}$$

*where* $\mu_{k}=\beta_{k}+1$ *and* $\nu_{k}=1+\frac{1}{\beta_{k}}$;

(ii) $R\neq\frac{1}{1-\alpha}$, $\beta_{k}>\frac{1}{2}$, *and for* $k=1, 2, \ldots, n$,

$$\begin{aligned}& (\mathrm{P}_{4})\quad \prod_{k=1}^{n} \frac{c_{k}}{2^{\beta_{k}}\Gamma(\beta_{k})}\biggl\{ \frac {\Gamma(2\beta_{k}-1)}{(1-\alpha)R-1} \bigl[1-e^{2(t_{k}-t_{k-1})(1-(1-\alpha)R)}\bigr]\biggr\} ^{\frac{1}{2}}< e^{-(1-\alpha)RT}, \\& (\mathrm{P}_{5})\quad \frac{c_{k}}{\Gamma(\beta_{k})}\biggl(\frac{e^{2t_{k}}\Gamma(2\beta _{k}-1)}{2^{2\beta_{k}-1}} \biggr)^{\frac{1}{2}} \\& \hphantom{(\mathrm{P}_{5})\quad}\quad {}\times\biggl( \int^{t_{k}} _{t_{k-1}}e^{2((1-\alpha)R-1)s}\biggl\{ \int ^{s}_{t_{k-1}}(1-\alpha)a(\nu)e^{-(1-\alpha)R\nu}\,d \nu\biggr\} ^{2} \,ds\biggr)^{\frac {1}{2}}\leq b_{k}, \end{aligned}$$

*and* ($\mathrm{P}_{3}$) *holds*;

(iii) $R=\frac{1}{1-\alpha}$ *and for* $k=1, 2, \ldots, n$,

$$\begin{aligned}& (\mathrm{P}_{6})\quad \prod_{k=1}^{n} \frac{c_{k}}{\Gamma(\beta_{k})}\biggl(\frac{\Gamma(\beta _{k}^{2})}{\mu_{k}^{\beta_{k}^{2}}}\biggr)^{\frac{1}{\mu_{k}}} (t_{k}-t_{k-1})^{\frac{1}{\nu_{k}}}< e^{-T}, \\& (\mathrm{P}_{7})\quad \frac{c_{k}}{\Gamma(\beta_{k})}\biggl(\frac{e^{\mu_{k}t_{k}}\Gamma(\beta _{k}^{2})}{\mu_{k}^{\beta_{k}^{2}}} \biggr)^{\frac{1}{\mu_{k}}} \biggl( \int^{t_{k}} _{t_{k-1}}\biggl\{ \int^{s}_{t_{k-1}}(1-\alpha)a(\nu)e^{-\nu }\,d \nu\biggr\} ^{\nu_{k}} \,ds\biggr)^{\frac{1}{\nu_{k}}}\leq b_{k}, \\& (\mathrm{P}_{8})\quad \theta\leq(1-\alpha) \int_{t_{n}}^{T}a(s)e^{(T-s)}\,ds; \end{aligned}$$

(iv) $R=\frac{1}{1-\alpha}$, $\beta_{k}>\frac{1}{2}$, *and for* $k=1, 2, \ldots, n$,

$$\begin{aligned}& (\mathrm{P}_{9})\quad \prod_{k=1}^{n} \frac{c_{k}}{\Gamma(\beta_{k})}\biggl(\frac{\Gamma(2\beta _{k}-1)(t_{k}-t_{k-1})}{2^{2\beta_{k}-1}}\biggr)^{\frac{1}{2}}< e^{-T}, \\& (\mathrm{P}_{10})\quad \frac{c_{k}}{\Gamma(\beta_{k})}\biggl(\frac{e^{2t_{k}}\Gamma(2\beta _{k}-1)}{2^{2\beta_{k}-1}} \biggr)^{\frac{1}{2}} \biggl( \int^{t_{k}} _{t_{k-1}}\biggl\{ \int^{s}_{t_{k-1}}(1-\alpha)a(\nu)e^{-\nu }\,d \nu\biggr\} ^{2}\,ds\biggr)^{\frac{1}{2}}\leq b_{k}, \end{aligned}$$

*and* ($\mathrm{P}_{8}$) *holds*. *Then we get* $y(t)\leq0$ *for* $t\in[0, T]$.

### Proof

Firstly, we use Case I(i) of Corollary 2.4 to prove (i). For $t\in[0, T]$, we have

$$\begin{aligned} y(t) \leq& \biggl\{ y^{1-\alpha}(0) \biggl(\prod _{t_{0}< t_{k}< t}\widetilde {A_{k}}\biggr)e^{R(1-\alpha)t}+ \sum_{t_{0}< t_{k}< t}\biggl(\prod_{t_{k}< t_{j}< t} \widetilde{A_{j}}\biggr)\widetilde{B_{k}}e^{R(1-\alpha)(t-t_{k})} \\ &{}- \int^{t}_{t_{l}}(1-\alpha)a(s)e^{R(1-\alpha)(t-s)}\,ds \biggr\} ^{\frac {1}{1-\alpha}}, \end{aligned}$$

where

$$\begin{aligned}& \widetilde{A_{k}}=\frac{c_{k}}{\Gamma(\beta_{k})}\biggl(\frac{\Gamma(\beta _{k}^{2})}{\mu_{k}^{\beta_{k}^{2}}} \biggr)^{\frac{1}{\mu_{k}}} \biggl(\frac{1-e^{\nu_{k}(t_{k}-t_{k-1})(1-(1-\alpha)R)}}{\nu_{k}((1-\alpha )R-1)}\biggr)^{\frac{1}{\nu_{k}}}, \\& \begin{aligned} \widetilde{B_{k}}&=\frac{c_{k}}{\Gamma(\beta_{k})}\biggl(\frac{e^{\mu _{k}t_{k}}\Gamma(\beta_{k}^{2})}{\mu_{k}^{\beta_{k}^{2}}} \biggr)^{\frac{1}{\mu_{k}}} \\ &\quad {}\times\biggl( \int^{t_{k}} _{t_{k-1}}e^{\nu_{k}((1-\alpha)R-1)s}\biggl\{ - \int ^{s}_{t_{k-1}}(1-\alpha)a(\nu)e^{-(1-\alpha)R\nu}\,d \nu\biggr\} ^{\nu_{k}} \,ds\biggr)^{\frac{1}{\nu_{k}}}-b_{k}. \end{aligned} \end{aligned}$$

It is obvious that $\widetilde{A_{k}}\geq0$ for all $k=1, 2, \ldots, n$. In fact, for the case of $(1-\alpha)R>1$ and $(1-\alpha)R\leq1$, both the denominator and the numerator in $\widetilde{A_{k}}$ have the same sign, hence we get $\widetilde{A_{k}}\geq0$. The condition ($\mathrm{P}_{2}$) implies that $\widetilde{B_{k}}\geq0$ for all $k=1, 2,\ldots n$. Then it is easy to show that $y(0)\leq0$. In fact, for $t=T$, we have

$$\begin{aligned} y(T) \leq& \biggl\{ y^{1-\alpha}(0) \biggl(\prod _{t_{0}< t_{k}< t}\widetilde {A_{k}}\biggr)e^{R(1-\alpha)T}+ \sum_{t_{0}< t_{k}< T}\biggl(\prod_{t_{k}< t_{j}< T} \widetilde{A_{j}}\biggr)\widetilde{B_{k}}e^{R(1-\alpha)(T-t_{k})} \\ &{}- \int^{T}_{t_{n}}(1-\alpha)a(s)e^{R(1-\alpha)(T-s)}\,ds \biggr\} ^{\frac {1}{1-\alpha}}, \end{aligned}$$

that is to say,

$$\begin{aligned} y(T)^{1-\alpha} \leq& y^{1-\alpha}(0) \Biggl(\prod _{k=1 }^{n}\widetilde {A_{k}} \Biggr)e^{R(1-\alpha)T}+\sum_{t_{0}< t_{k}< T}\biggl(\prod _{t_{k}< t_{j}< T} \widetilde{A_{j}}\biggr) \widetilde{B_{k}}e^{R(1-\alpha)(T-t_{k})} \\ &{}- \int^{T}_{t_{n}}(1-\alpha)a(s)e^{R(1-\alpha)(T-s)}\,ds. \end{aligned}$$

Using the hypothesis $y^{1-\alpha}(0)=y^{1-\alpha}(T)+\theta$ and ($\mathrm{P}_{1}$), ($\mathrm{P}_{2}$), we get

$$\begin{aligned}& y^{1-\alpha}(0)\Biggl[1-\Biggl(\prod_{k=1 }^{n} \widetilde{A_{k}}\Biggr)e^{R(1-\alpha)T}\Biggr] \\& \quad \leq\theta+\sum_{t_{0}< t_{k}< T}\biggl(\prod _{t_{k}< t_{j}< T} \widetilde{A_{j}}\biggr) \widetilde{B_{k}}e^{R(1-\alpha)(T-t_{k})} - \int^{T}_{t_{n}}(1-\alpha)a(s)e^{R(1-\alpha)(T-s)}\,ds \\& \quad \leq0, \end{aligned}$$

which implies that $y^{1-\alpha}(0)\leq0$, since $0<\alpha<1$, we get $y(0)\leq0$.

To prove (ii), applying Case I(ii) of Corollary 2.4 for $t\in[0, T]$, we have

$$\begin{aligned} y(t) \leq& \biggl\{ y^{1-\alpha}(0) \biggl(\prod _{t_{0}< t_{k}< t}\widetilde {C_{k}}\biggr)e^{R(1-\alpha)t}+ \sum_{t_{0}< t_{k}< t}\biggl(\prod_{t_{k}< t_{j}< t} \widetilde{C_{j}}\biggr)\widetilde{D_{k}}e^{R(1-\alpha)(t-t_{k})} \\ &{}- \int^{t}_{t_{l}}(1-\alpha)a(s)e^{R(1-\alpha)(t-s)}\,ds \biggr\} ^{\frac {1}{1-\alpha}}, \end{aligned}$$

where

$$\begin{aligned}& \widetilde{C_{k}}=\frac{c_{k}}{2^{\beta_{k}}\Gamma(\beta_{k})}\biggl\{ \frac {\Gamma(2\beta_{k}-1)}{(1-\alpha)p-1} \bigl[1-e^{2(t_{k}-t_{k-1})(1-(1-\alpha)p)}\bigr]\biggr\} ^{\frac{1}{2}}, \\& \begin{aligned} \widetilde{D_{k}}&=\frac{c_{k}}{\Gamma(\beta_{k})}\biggl(\frac{e^{2t_{k}}\Gamma (2\beta_{k}-1)}{2^{2\beta_{k}-1}} \biggr)^{\frac{1}{2}} \biggl( \int^{t_{k}} _{t_{k-1}}e^{2((1-\alpha)p-1)s}\biggl\{ \int ^{s}_{t_{k-1}}(1-\alpha)q(\nu)e^{-(1-\alpha)p\nu}\,d \nu\biggr\} ^{2} \,ds\biggr)^{\frac {1}{2}} \\ &\quad {}-b_{k}. \end{aligned} \end{aligned}$$

Then using a similar method to proof of Case I(i) with conditions ($\mathrm{P}_{1}$) and ($\mathrm{P}_{3}$), we deduce $y(0)\leq0$.

Next, we prove (iii). Applying Case II(i) of Corollary 2.4 for $t\in [0, T]$, we have

$$\begin{aligned} y(t) \leq& \biggl\{ y^{1-\alpha}(0) \biggl(\prod _{t_{0}< t_{k}< t}\widetilde {E_{k}}\biggr)e^{t}+ \sum_{t_{0}< t_{k}< t}\biggl(\prod_{t_{k}< t_{j}< t} \widetilde{E_{j}}\biggr)\widetilde{F_{k}}e^{(t-t_{k})} \\ &{}- \int^{t}_{t_{l}}(1-\alpha)a(s)e^{(t-s)}\,ds \biggr\} ^{\frac{1}{1-\alpha}}, \end{aligned}$$

where

$$\begin{aligned}& \widetilde{E_{k}}=\frac{c_{k}}{\Gamma(\beta_{k})}\biggl(\frac{\Gamma(\beta _{k}^{2})}{\mu_{k}^{\beta_{k}^{2}}} \biggr)^{\frac{1}{\mu_{k}}} (t_{k}-t_{k-1})^{\frac{1}{\nu_{k}}}, \\& \widetilde{F_{k}}=\frac{c_{k}}{\Gamma(\beta_{k})}\biggl(\frac{e^{\mu _{k}t_{k}}\Gamma(\beta_{k}^{2})}{\mu_{k}^{\beta_{k}^{2}}} \biggr)^{\frac{1}{\mu_{k}}} \biggl( \int^{t_{k}} _{t_{k-1}}\biggl\{ \int^{s}_{t_{k-1}}(1-\alpha)q(\nu)e^{-\nu }\,d \nu\biggr\} ^{\nu_{k}} \,ds\biggr)^{\frac{1}{\nu_{k}}}-b_{k}. \end{aligned}$$

It is easy to see that $\widetilde{E_{k}}\geq0$ and $\widetilde {F_{k}}\leq0$ for all $k=1, 2, \ldots, n$. Then using a similar method to proof (i) with conditions ($\mathrm{P}_{6}$) and ($\mathrm{P}_{7}$), it is easy to show that $y(0)\leq0$.

Similarly, to prove (iv), we apply Case II(ii) of Corollary 2.4 for $t\in[0, T]$, we get

3.1 $$\begin{aligned} m(t) \leq& \biggl\{ m^{1-\alpha}(t_{0}) \biggl(\prod _{t_{0}< t_{k}< t}\widetilde {G_{k}}\biggr)e^{(t-t_{0})}+ \sum_{t_{0}< t_{k}< t}\biggl(\prod_{t_{k}< t_{j}< t} \widetilde{G_{j}}\biggr)\widetilde{H_{k}}e^{(t-t_{k})} \\ &{}+ \int^{t}_{t_{l}}(1-\alpha)q(s)e^{(t-s)}\,ds \biggr\} ^{\frac{1}{1-\alpha}}, \end{aligned}$$

where

$$\begin{aligned}& \widetilde{G_{k}}=\frac{c_{k}}{\Gamma(\beta_{k})}\biggl(\frac{\Gamma(2\beta _{k}-1)(t_{k}-t_{k-1})}{2^{2\beta_{k}-1}} \biggr)^{\frac{1}{2}}, \\& \widetilde{H_{k}}=\frac{c_{k}}{\Gamma(\beta_{k})}\biggl(\frac{e^{2t_{k}}\Gamma (2\beta_{k}-1)}{2^{2\beta_{k}-1}} \biggr)^{\frac{1}{2}} \biggl( \int^{t_{k}} _{t_{k-1}}\biggl\{ \int^{s}_{t_{k-1}}(1-\alpha)q(\nu)e^{-\nu }\,d \nu\biggr\} ^{2}\,ds\biggr)^{\frac{1}{2}}+b_{k}. \end{aligned}$$

Then using a similar method as that of (iii), it is easy to show that $y(0)\leq0$. This completes the proof. □

### Example 3.3

Let $x\in PC^{1}[\mathbb{R}_{+}, \mathbb{R}]$, and for $k=1, 2, \ldots$ , we suppose

3.2 $$ \textstyle\begin{cases} x'(t)=f(t, x(t)),\quad t\neq t_{k}, t\in[t_{0}, \infty), \\ \Delta x(t_{k})=X_{k} ((I_{t_{k-1}}^{\beta_{k}}x)(t_{k})), \\ x(t_{0})=x_{0}, \end{cases} $$

where $f\in C(\mathbb{R}_{+}\times\mathbb{R}, \mathbb{R})$, $X_{k}\in C(\mathbb{R}, \mathbb{R})$, $0\leq t_{0}< t_{1}< t_{2}<\cdots$, $\lim_{k\rightarrow\infty}t_{k}=\infty$, $\Delta x(t_{k})=x(t_{k}^{+})-x(t_{k})$, $\beta_{k}>0$ ($k=1, 2, \ldots$) and $x_{0}$ are constants. Assume there exists a constant $L>0$, such that

3.3 $$ \bigl\vert f\bigl(t, x(t)\bigr) \bigr\vert \leq L \bigl\vert x(t) \bigr\vert ^{\alpha}\quad \mbox{for } t\geq t_{0}, $$

and there exists a constant $M_{k}>0$, such that

3.4 $$ \bigl\vert X_{k}(x) \bigr\vert \leq M_{k} \vert x \vert ,\quad x\in\mathbb{R}, k=1, 2, \ldots. $$

Then, for $t\geq t_{0}$, the following inequalities hold:

3.5 $$\begin{aligned} \bigl\vert x(t) \bigr\vert \leq& \biggl\{ \biggl\{ x_{0}\prod _{t_{0}< t_{k}< t}\widetilde{R_{k}}+\sum _{t_{0}< t_{k}< t}\biggl[\prod_{t_{k}< t_{j}< t} \widetilde{R_{j}}\biggr]\widetilde {S_{k}}\biggr\} ^{1-\alpha} \\ &{}+(1-\alpha)2^{(k-1)\alpha}L(t-t_{0})\biggr\} ^{\frac{1}{1-\alpha}}, \end{aligned}$$

where

$$\begin{aligned}& \widetilde{R_{k}}=2^{\frac{\alpha}{1-\alpha}}\biggl[1+\frac{M_{k}}{\Gamma (\beta_{k})} \int_{t_{k-1}}^{t_{k}}(t_{k}-s)^{\beta_{k}-1} \,ds\biggr], \\& \widetilde{S_{k}}=\frac{M_{k}}{\Gamma(\beta_{k})}\frac{1-\alpha }{2-\alpha}2^{\frac{(k-1)\alpha}{1-\alpha}} \bigl(L(1-\alpha)\bigr)^{\frac {1}{1-\alpha}} \bigl[(t_{k}-t_{0})^{\frac{2-\alpha}{1-\alpha}}-(t_{k-1}-t_{0})^{\frac {2-\alpha}{1-\alpha}} \bigr]. \end{aligned}$$

### Proof

Suppose $x=x(t)$ is a solution of (3.2), we integrate this equation to obtain

$$x(t)=x(t_{0})+ \int^{t}_{t_{0}}f\bigl(s, x(s)\bigr)\,ds+\sum _{t_{0}< t_{k}< t}X_{k} \bigl(\bigl(I_{t_{k-1}}^{\beta_{k}}x \bigr) (t_{k})\bigr), $$

then (3.3) and (3.4) imply that

$$\begin{aligned} \bigl\vert x(t) \bigr\vert \leq& \bigl\vert x(t_{0}) \bigr\vert + \int^{t}_{t_{0}} \bigl\vert f\bigl(s, x(s)\bigr) \bigr\vert \,ds+\sum_{t_{0}< t_{k}< t} \bigl\vert X_{k} \bigl(\bigl(I_{t_{k-1}}^{\beta_{k}}x\bigr) (t_{k})\bigr) \bigr\vert \\ \leq& \vert x_{0} \vert + \int_{t_{0}} ^{t}L \bigl\vert x(s) \bigr\vert ^{\alpha}\,ds+\sum_{t_{0}< t_{k}< t}M_{k} \bigl\vert \bigl(I_{t_{k-1}}^{\beta_{k}}x\bigr) (t_{k}) \bigr\vert . \end{aligned}$$

Then Theorem 2.7 yields the estimate of (3.5), and

$$\begin{aligned} \widetilde{S_{k}} =&{M_{k}} {\Gamma( \beta_{k})}2^{\frac{(k-1)\alpha }{1-\alpha}}(1-\alpha)^{\frac{1}{1-\alpha}} \int_{t_{k-1}}^{t_{k}}\biggl( \int ^{s}_{t_{0}} Ld\nu\biggr)^{\frac{1}{1-\alpha}}\,ds \\ =&\frac{M_{k}}{\Gamma(\beta_{k})}\frac{1-\alpha}{2-\alpha}2^{\frac {(k-1)\alpha}{1-\alpha}}\bigl(L(1-\alpha) \bigr)^{\frac{1}{1-\alpha}} \bigl[(t_{k}-t_{0})^{\frac{2-\alpha}{1-\alpha}}-(t_{k-1}-t_{0})^{\frac {2-\alpha}{1-\alpha}} \bigr]. \end{aligned}$$

 □

As a special case, we consider the following initial value problem of impulsive differential equation with finite discontinuous points.

### Example 3.4

Consider the initial value problem of the form

3.6 $$ \textstyle\begin{cases} x'(t)=f(x(t)),\quad t\in[{0}, \infty), t\neq t_{k}, 1\leq k\leq10, \\ \Delta x(t_{k})=(I_{{k-1}}^{\beta_{k}}x)(t_{k}), \\ x(0)=0, \end{cases} $$

where $f(x)= \big\{\scriptsize{\begin{array}{l@{\quad}l} 2\sqrt{|x|}, & |x|<1, \\ \sqrt[3]{x}+1, & |x|\geq1, \end{array}}$ $t_{k}=k$, $\beta_{k}=\frac{1}{k+1}$ for $1\leq k \leq10$. In this case, we see that $|f(x)|\leq2\sqrt{|x|}$ with $L=2$ and $\alpha=\frac{1}{2}$, and $|X_{k}(x)|=|x|$ with $M_{k}=1$. By direct calculation, we get

$$\begin{aligned}& \widetilde{R_{k}}=2\biggl[1+\frac{1}{\Gamma(\frac{1}{k+1})} \int _{k-1}^{k}({k}-s)^{\frac{1}{k+1}-1}\,ds\biggr]=2 \biggl[1+\frac{k+1}{\Gamma(\frac{1}{k+1})}\biggr]=2+\frac{2}{\Gamma(\frac{k+2}{k+1})}, \\& \widetilde{S_{k}}=\frac{1}{\Gamma(\frac{1}{k+1})}\frac{1}{3} 2^{k-1}\bigl[k^{3}-(k-1)^{3}\bigr]= \frac{2^{k-1}}{\Gamma(\frac{1}{k+1})}\biggl[k^{2}-k+\frac{1}{3}\biggr]. \end{aligned}$$

Then the solution of the initial value problem (3.6) can be estimated as

3.7 $$ \bigl\vert x(t) \bigr\vert \leq \biggl\{ \biggl(\sum _{{0}< k< t}\biggl[\prod_{k< j< t}\biggl(2+ \frac {2}{\Gamma(\frac{j+2}{j+1})}\biggr)\biggr]\frac{2^{k-1}}{\Gamma(\frac{1}{k+1})}\biggl[k^{2}-k+ \frac{1}{3}\biggr] \biggr)^{1/2} +2^{\frac{k-1}{2}}t \biggr\} ^{2}. $$

Moreover, for $t\geq10$, we have

$$\bigl\vert x(t) \bigr\vert \leq\bigl(c^{*}+16\sqrt{2}t\bigr)^{2} $$

for some constant calculated through (3.7) with $k=10$.
